# Highly Conductive Liquid Metal Emulsion Gels for Three‐Dimensionally Printed Stretchable Electronics

**DOI:** 10.1002/advs.202503449

**Published:** 2025-07-06

**Authors:** Qianying Lu, Ting Fang, Chenyang Ye, Yanyan Li, Ming Wu, Yuping Sun, Desheng Kong, Xiaoliang Wang, Yan‐qing Lu

**Affiliations:** ^1^ State Key Laboratory of Analytical Chemistry for Life Science and Jiangsu Key Laboratory of Artificial Functional Materials Nanjing University Nanjing 210021 China; ^2^ College of Engineering and Applied Sciences National Laboratory of Solid State Microstructures and Collaborative Innovation Center of Advanced Microstructures Nanjing University Nanjing 210093 China; ^3^ Key Laboratory of High Performance Polymer Materials and Technology of Ministry of Education Department of Polymer Science and Engineering School of Chemistry and Chemical Engineering Nanjing University Nanjing 210023 China; ^4^ Key Laboratory of Intelligent Optical Sensing and Manipulation Nanjing University Nanjing 210093 China

**Keywords:** 3D printing, liquid metal, printable electronics, stretchable conductors, stretchable electronics

## Abstract

Gallium‐based liquid metals are promising for stretchable electronics due to their inherent deformability and excellent conductivity. However, the lack of a scalable and automated fabrication process has limited their practical applications. This study introduces a two‐step method to create liquid metal emulsion gels suitable for 3D printing, characterized by densely packed liquid metal microcapsules within polymer matrices. The resulting emulsion gel demonstrates favorable rheological properties for 3D printing and minimal shrinkage through solidification. With a substantial fraction of sizable microcapsules, the printed features can be activated into compliant conductors with exceptional conductivity of ≈2.2× 10^4^ S cm^−1^ and an ultrahigh stretchability of up to ≈1000% strain. Stretchable lighting emitting diode displays and near field communication tags are successfully fabricated through 3D printing to demonstrate the practicality of liquid metal microcapsule gels. These developments provide a versatile platform to design liquid metal inks for printed stretchable electronics.

## Graphical abstract

This study presents a scalable two‐step method to create liquid metal emulsion gels suitable for 3D printing. The emulsion gel contains densely packed microcapsules within polymer matrices, resulting in minimal shrinkage and optimal printability. Printed features achieve excellent conductivity and ultrahigh deformability after mechanical activation, facilitating the automated manufacturing of diverse stretchable electronic devices.

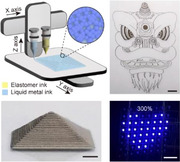

## 1 Introduction

The advancement of electronic technology has traditionally emphasized processing speed and energy efficiency. However, the rise of stretchable electronics represents a new era characterized by mechanical adaptability, particularly well‐suited for next‐generation wearables.^[^ ^1^, ^2^, ^3^ ^]^ The deformable forms of devices enable seamless and reliable integration with the human body, presenting promising prospects in health monitoring,^[^ ^4^, ^5^ ^]^ human–machine interfaces,^[^ ^6^ ^]^ and advanced prosthetics.^[^ ^7^, ^8^ ^]^ Compliant conductors are essential building materials for stretchable electronic devices, finding diverse applications in active electrodes and electronic interconnects.^[^ ^9^ ^]^ Solid conductors such as conducting polymers and composites comprise deformable conductive networks within polymer matrices.^[^ ^10^, ^11^ ^]^ Despite decent conductivity, these materials struggle to withstand large and repetitive tensile strains due to cumulative damage to the conductive network.^[^ ^12^, ^13^ ^]^ In contrast, liquid‐state materials, particularly gallium‐based alloys known as liquid metals, exhibit ideal deformability after being incorporated into elastomer matrices, limited only by the fracture strain of these elastomers.^[^ ^14^ ^]^ These liquid metals possess favorable attributes, including high conductivity (≈3.4 × 10^4^ S cm^−1^), low melting points, and biocompatibility, positioning them as an emerging material category for compliant conductors.^[^ ^15^, ^16^ ^]^

The development of stretchable electronics heavily relies on the accessibility to conductive liquid metal features. One straightforward approach involves filling liquid metal into microfluidic channels embedded in elastomers.^[^ ^17^ ^]^ However, this method is constrained by the intricate fabrication of soft microfluidic devices and its incompatibility with advanced circuit designs. Alternatively, various liquid metal patterning techniques have been extensively explored, such as microcontact printing,^[^ ^18^ ^]^ selective wetting,^[^ ^19^, ^20^ ^]^ laser structuring,^[^ ^21^ ^]^ and photolithography.^[^ ^22^, ^23^ ^]^ Another emerging fabrication approach for stretchable devices and circuits is extrusion‐based 3D printing, allowing the integration of multiple functional electronic materials in an automated sequence.^[^ ^24^, ^25^ ^]^ Nevertheless, direct printing of liquid metals is often challenging due to their low viscosity and high surface tension.^[^ ^26^, ^27^ ^]^ As an alternative, liquid metals can be mechanically mixed with a polymer solution or precursor, producing nano or microsized droplets dispersed within the polymer matrix.^[^ ^28^, ^29^ ^]^ These liquid metal droplets are encapsulated by spontaneously formed native oxides, forming core–shell capsules.^[^ ^30^, ^31^ ^]^ Nonetheless, this mixing technique poses challenges in preparing stable emulsions with a high liquid metal percentage, mainly due to the rapid coalescence of the formed capsules.^[^ ^32^ ^]^ Recently, an in situ‐formed hydrogel layer has been introduced to stabilize liquid metal capsules, allowing for a significantly increased liquid metal percentage in the resulting mixtures.^[^ ^33^ ^]^ However, it is essential to note that this mechanically tough hydrogel restricts the formation of a highly conductive network within printed composites, undermining its potential benefits for electronic device applications.

The 3D‐printed features are typically insulating composites that contain isolated liquid metal capsules. These composites require additional activation through stretching,^[^ ^34^ ^]^ compression,^[^ ^28^ ^]^ or laser irradiation^[^ ^35^ ^]^ to release the enclosed liquid metal and establish electrical conductivity. As the capsules are surrounded by the polymer matrix, a higher fraction of the liquid metal in these composites is beneficial for effective activation. Consequently, extensive research has focused on developing ink dispersions with a higher percentage of liquid metals.^[^ ^36^ ^]^ Moreover, the size of the liquid metal capsules affects the ink dispersion and subsequent activation.^[^ ^37^ ^]^ However, the mixing parameters simultaneously affect the size and uniformity of the liquid metal capsules,^[^ ^32^ ^]^ making it challenging to create ink dispersions with customized compositions. As a result, the level of conductivity achieved in 3D‐printed liquid metal composites is significantly lower than that of their bulk counterparts.

Here, we present the development of liquid metal emulsion gels for extrusion‐based 3D printing. The two‐step preparation involves the controlled synthesis of liquid metal microcapsules, followed by their combination with elastomers to produce tailored ink dispersions. When the liquid metal microcapsules exceed a critical volume threshold for random close packing, this mixture transitions into a gel state without requiring extra additives. These emulsion gels exhibit favorable rheological properties, including shear‐thinning responses and yield flow behaviors, which enable the facile creation of diverse 2D patterns and 3D features. The formation mechanism relies on the unique preparation process to achieve the densely packed microstructure, which is broadly applicable across various microcapsule sizes and polymer matrices. Moreover, the packed microcapsules support excellent structural retention of the printed feature, resulting in a minimal shrinkage of less than 4% after solvent evaporation. With a substantial fraction of sizable liquid microcapsules, the microcapsule composite attains a high conductivity of ≈2.2×10^4^ S cm^−1^ after peeling activation, corresponding to ≈65% of the bulk metal. The activated liquid metal conductor can stretch up to 1000% strain and endure repetitive deformations. To showcase practical demonstrations, the liquid metal emulsion gel is combined with elastomer ink to create stretchable electronic devices, such as stretchable light emitting diode (LED) displays and near field communication (NFC) tags, using a multimaterial 3D printing approach. By leveraging the layer‐by‐layer stacking capability of 3D printing, multiple layers of the liquid metal composite can be easily deposited to minimize ohmic losses, achieving a markedly low sheet resistance of ≈0.5 mΩ sq.^−1^ in high aspect ratio tracks. The developments outlined here provide a robust strategy for formulating liquid metal‐based inks to construct 3D‐printed stretchable electronics.

## 2 Results and Discussion

### 2.1 Preparation of Liquid Metal Emulsion Gels

**Figure 1:**
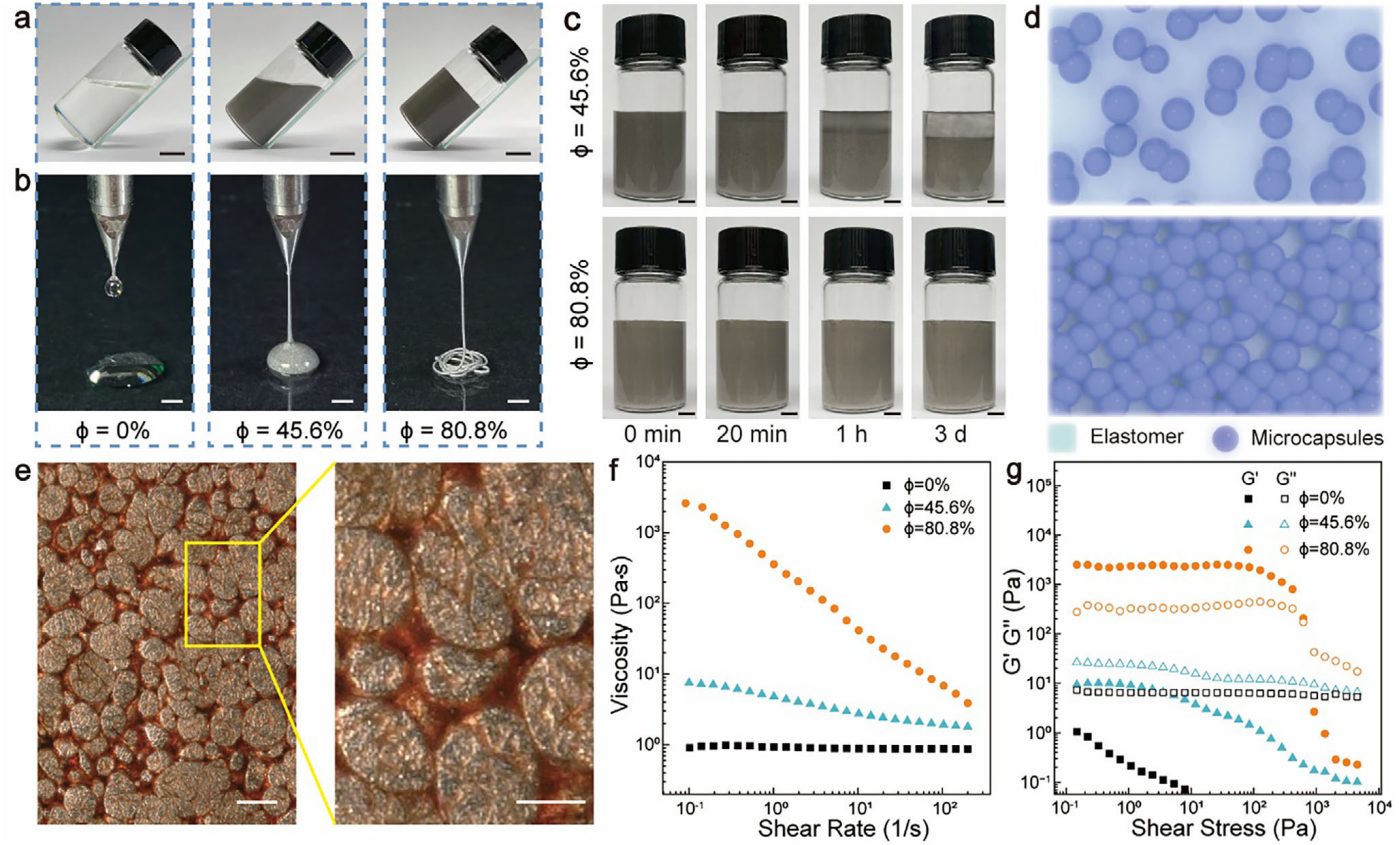
Preparation of liquid metal emulsion gels. a) Liquid metal microcapsule mixture with SIS elastomer solution at a volume concentration (ϕ) of 0% (left), 45.6% (medium), and 80.8% (right) inside an inclined vial. Scale bars: 1 cm. b) Corresponding mixtures extruded through a dispensing needle with a 200 µm orifice. Scale bars: 2 mm. c) Liquid metal/SIS solution mixtures at 45.6% (up) and 80.8% (down) at different settling durations. Scale bars: 5 mm. d) Schematic illustration of liquid metal microcapsule mixtures in dilute (up) and concentrated (down) states. e) Optical microscopy images displaying emulsion gels with densely packed liquid metal microcapsules, with the elastomer solution dyed in orange to facilitate visualization. Scale bars: 50 µm. f) Viscosity versus shear rate of liquid metal mixtures. g) Storage moduli (G′) and loss moduli (G″) as a function of shear stress.

The synthesis of liquid metal microcapsules follows a top‐down approach, involving the mechanical agitation of bulk alloys to generate microdroplets enclosed within self‐assembled fatty acid shells (refer to the Experimental Section for detailed procedures).^[^ ^38^ ^]^ Emulsification conducted for a duration of 30 s yields microcapsules measuring 108.6 µm in size (Figure S1, Supporting Information). These microcapsules are blended with a styrene−isoprene−styrene (SIS)/xylene solution to create inks. Initially, the addition of microcapsules does not alter the liquid properties of the SIS solution. However, the mixture turns into a gel state at a high volume percentage (ϕ), as depicted in **Figure** **1**a. In Figure 1b, the SIS solution is pushed out of a needle, forming separate droplets. A mixture containing 45.6% microcapsules creates a filamentary jet that merges into a puddle, indicating its viscous liquid nature. When the microcapsule content increases to 80.8%, the mixture generates stable filaments that have a gel‐like solid appearance. Additionally, a mixture containing a low concentration of liquid metal experiences storage stability issues,^[^ ^39^ ^]^ as evidenced by apparent layering within a few hours (Figure 1c). However, high‐loading liquid metal microcapsules allow the mixture to maintain a homogeneous state even after settling for several days. As schematically illustrated in Figure 1d, low‐percentage mixtures contain discrete and clustered microcapsules that cannot resist gravitational sedimentation. In contrast, high‐percentage mixtures form particulate gels with interconnected microcapsules, resulting in a stable and uniform dispersion. The microstructure of a representative emulsion gel is revealed by optical microscopy images in Figure 1e. Within the colored SIS solution matrix, liquid metal microcapsules form a dense packing state. The surface topographic image in Figure S2 (Supporting Information) further confirms that the microcapsules establish interconnected networks inside the emulsion gel.

The flow properties are analyzed through rheological measurements. Figure 1f and Figure S3 (Supporting Information) show the viscosity as a function of the shear rate. The SIS solution has a low viscosity(η) of ≈1.0 Pa·s that remains nearly constant regardless of the shear rate. By incorporating liquid metal microcapsules, the low‐shear viscosity of the mixtures increases, as shown in Figure S4 (Supporting Information), due to the microcapsule clustering to hinder the flow.^[^ ^40^, ^41^ ^]^ Specifically, the apparent viscosity at 0.1 s^−1^ is 7.5, 2606.0, and 3571.3 Pa·s for volume fractions of 45.6%, 80.8%, and 86.3%, respectively. All mixtures demonstrate shear‐thinning behaviors, displaying reduced viscosity with increasing shear rate. At shear rates relevant to extrusion‐based printing (≈10^2^ s^−1^),^[^ ^42^, ^43^ ^]^ the apparent viscosity decreases to a level within one order of magnitude of the base elastomer solution, facilitating smooth extrusion. Further insights into these mixtures are provided by the dynamic modulus profiles in Figure 1g and Figure S3 (Supporting Information). When microcapsules stay in low percentages, the mixture has a storage modulus (G') lower than loss modulus (G″) across the entire shear stress range, suggesting its liquid properties. However, once microcapsules exceed a threshold of 67.7%, the mixture shows a plateau in G' that surpasses G″ at low shear stresses, signifying the solid behavior due to the formation of an emulsion gel. The moduli curves also reveal a crossover point that defines the shear yield stress (τ_y_), beyond which the mixture transitions into a liquid state. This volume threshold for emulsion gel formation closely aligns with the theoretical value for random close packing in monodispersed particles (≈64%).^[^ ^44^ ^]^ In these emulsion gels, both the storage modulus and yield stress increase with the microcapsule volume fraction, as shown in Figure S4 (Supporting Information), enabling the printed feature to maintain its structure by resisting flow and collapse. Additionally, inks formulated using liquid metal microcapsules with oxide shells at high volume concentrations also exhibit rheological properties of yield stress fluids (see Figure S5, Supporting Information), which further confirms the formation of emulsion gels through the densely packed microstructure.

### 2.2 3D Printing of Liquid Metal Emulsion Gels

**Figure 2:**
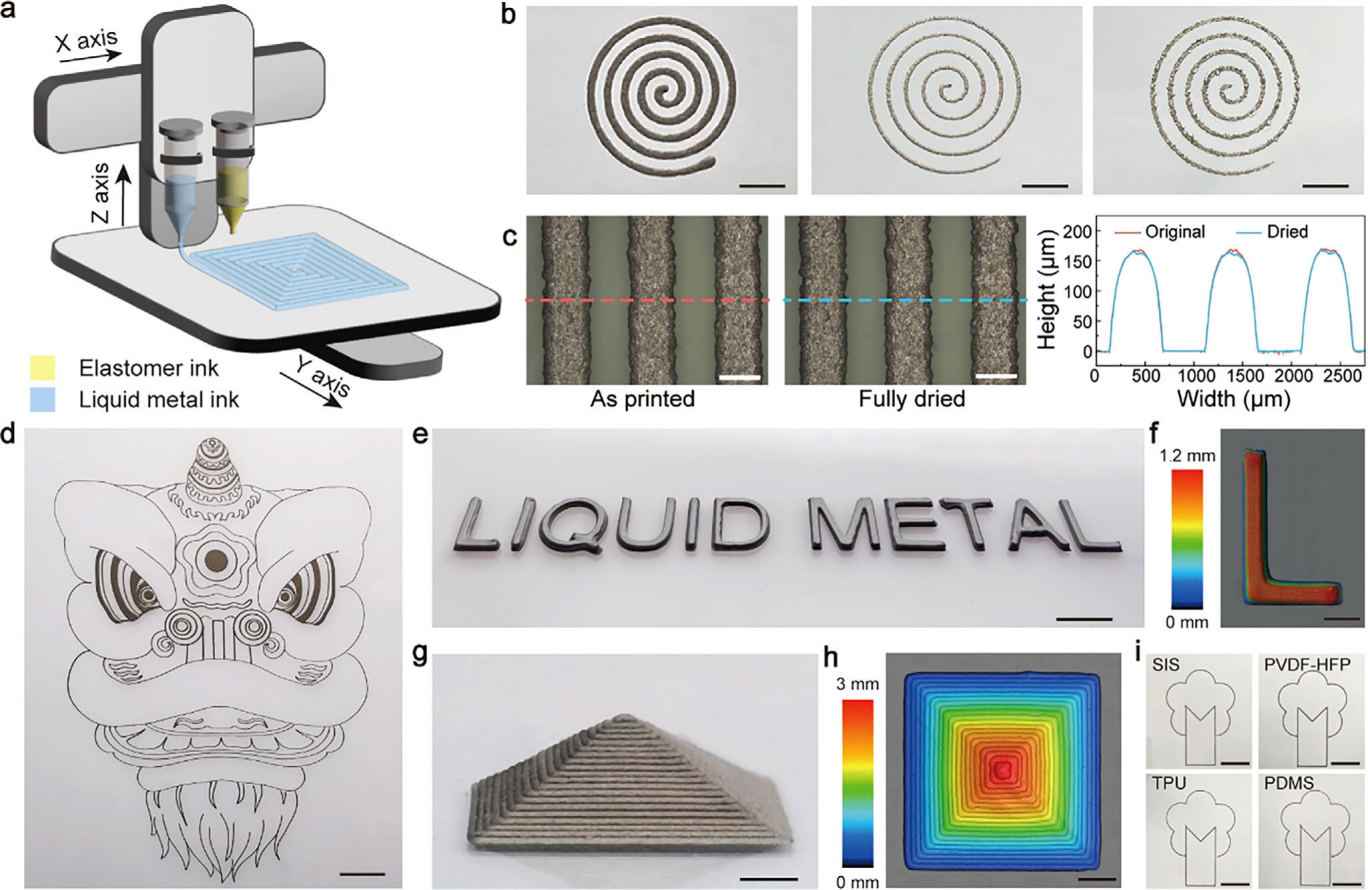
Extrusion‐based 3D printing using liquid metal emulsion gels. a) Schematic experimental set‐up of extrusion‐based 3D printing. b) Spiral‐shaped patterns generated by liquid metal/SIS solution mixtures at 45.6% (left), 80.8% (middle), and 86.3% (right). Scale bars: 3 mm. c) Optical microscopy images and corresponding height profiles of dashed linecuts showing single‐layer line‐shaped tracks in the original and thoroughly dried states. Scale bars: 500 µm. d) Optical image of a printed Chinese lion sketch showcasing the capability to create intricate patterns. Scale bar: 20 mm. e) Optical image of a “LIQUID METAL” shaped feature formed by stacking multiple print layers. Scale bar: 10 mm. f) Corresponding topographic profile of the letter “L”. Scale bar: 3 mm. g) Optical image showing a 3D miniaturized pyramid structure. Scale bar: 200 µm. h) Corresponding surface topographic image. Scale bar: 2 mm. i) Tree‐shaped patterns printed using liquid metal emulsion gels containing different elastomers, including SIS, TPU, PDMS, and PVDF‐HFP. Scale bars: 1 cm.

Extrusion‐based 3D printing allows direct fabrication of complex circuit patterns on target substrates, as schematically illustrated in **Figure** **2**a. The functional inks loaded in syringes are mounted on a computer‐controlled, motorized three‐axis stage. The inks are then pneumatically extruded through dispensing needles into filamentary forms, building the desired features layer‐by‐layer following programmed stage movements. Both liquid metal and elastomer inks are necessary for creating circuit patterns in multilayer designs. The composition of liquid metal‐based inks directly impacts their printing outcome. A liquid‐state mixture (ϕ = 45.6%) can be dispensed at a low pressure of ≈30 kPa, as depicted in Figure 2b. However, the significant spreading of the extruded mixture leads to distorted patterns. Conversely, an emulsion gel mixture with ϕ = 80.8% can be stably extruded into fine filaments at an increased pressure of ≈150 kPa, producing high‐fidelity features such as the sharp spiral pattern. Increasing the microcapsule content to 86.3% still allows for extrusion into fine filaments, but the presence of liquid metal beads on the filament indicates uncontrolled ruptures of microcapsules during printing. Therefore, an emulsion gel with ϕ = 80.8% is considered the optimal choice for most experiments unless otherwise specified. Notably, the elastomer solution addition is critical for stabilizing the liquid metal emulsion gel during printing. When pure liquid metal microcapsules are extruded from the dispensing needles, the rupture of the fatty acid shell causes the microcapsules to coalesce into large droplets, preventing the formation of well‐defined features (see Figure S6, Supporting Information).

The solidification of printed emulsion gels relies on solvent evaporation, accelerated by heating the stage. The representative striped features through the single‐layer print maintain their original morphology upon solidification, as depicted in Figure 2c. This drying process results in minimal shrinkage, measuring below 5%. In contrast, solution‐processed composites often experience significant volume changes during solvent evaporation, reaching up to 14.3% for this mixture (ϕ = 80.8%) under the assumption of uniform shrinkage (Figure S7, Supporting Information). The significant shrinkage poses challenges for 3D printed parts, leading to mechanical stress and structural distortion.^[^ ^45^, ^46^ ^]^ Moreover, the low shrinkage of the emulsion gel has been corroborated by additional testing in multilayer prints (Figures S8 and S9, Supporting Information) and solvents of higher boiling points (Figures S10 and S11, Supporting Information). The low volume change is attributed to the compact microcapsules within the gel, forming a sturdy framework that resists shrinkage. As a result, the printed emulsion gel can be directly dried into desired features while maintaining high structural fidelity. Notably, the liquid metal fraction increases further after solvent removal, which is beneficial for post‐activation aimed at enhancing conductivity.

Liquid metal emulsion gels are well‐suited for creating desired features. The optical image in Figure 2d displays a representative 2D feature of a Chinese lion sketch, showcasing the fabrication capability for complex patterns. Additionally, this additive manufacturing process facilitates the facile preparation of 3D objects through a layerwise build sequence. By stacking identical print layers, it is possible to form a “LIQUID METAL” feature with significantly enhanced height profiles, as depicted in Figure 2e,f and Figure S12 (Supporting Information). Another example involves a miniaturized pyramid structure with progressively reduced dimensions of the print layers (refer to Figure 2g,h). Movies S1 and S2 (Supporting Information) reveal the dynamic processes involved in creating these 3D features. These results effectively underscore the exceptional printability of liquid metal emulsion gels for extrusion‐based 3D printing.

The ink formulation strategy allows the convenient development of liquid metal mixtures with controlled compositions. Apart from the SIS solution, liquid metal microcapsules can be mixed with other polymer matrices, such as thermoplastic polyurethane (TPU) solution, poly(vinylidene fluoride‐hexafluoropropylene)(PVDF‐HFP) solution, or polydimethylsiloxane (PDMS) prepolymer. Rheological measurements of the resulting mixtures in Figures S14–S16 (Supporting Information) have confirmed the formation of emulsion gels at a high microcapsule loading of 80.8%. As shown by optical images in Figure 2i, these mixtures have suitable flow characteristics to allow the facile creation of tree‐like patterns. Beyond their different chemical compositions, these polymers exhibit distinct mechanical properties, as shown in Figure S13 (Supporting Information), indicating the potential to modulate the tensile responses of the printed composites. These findings illustrate a generic formation mechanism of the emulsion gel that can serve as a sturdy basis for preparing 3D printable liquid metal inks.

### 2.3 Mechanical Activation for Printed Liquid Metal Microcapsule Composites

**Figure 3:**
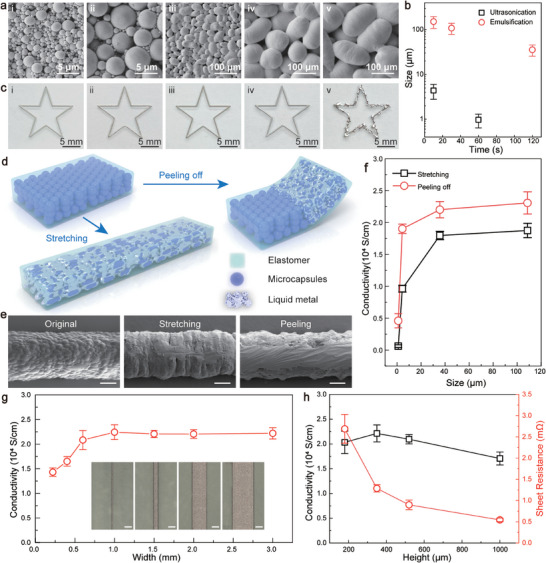
Mechanical activation of printed liquid metal microcapsule composites. a) SEM images of liquid metal microcapsules synthesized through sonication for 60 (i) and 10 (ii) s and through emulsification for 120 (iii), 30 (iv), and 10 (v) s. b) Microcapsule size obtained through different synthesis conditions. Data represents mean ± s.d. (n = 4). c) Star‐shaped patterns printed using emulsion gels with varying‐sized liquid metal microcapsules. d) Schematic illustration of the mechanical activation of liquid metal composites through stretching or peeling. e) SEM images revealing microstructural changes in the composite through activation. Scale bars: 200 µm. f) Conductivity of activated composites containing varying‐sized microcapsules. Data represents mean ± s.d. (n = 4). g) Conductivity of the activated composite with different widths after peeling activation. Inset: Optical microscopy images showing line‐shaped features of different widths. Scale bars: 1 mm. Data represents mean ± s.d. (n = 6). h) Conductivity and sheet resistance of the peeling activated composite with respect to the feature height. Data represents mean ± s.d. (n = 6).

Liquid metal/fatty acid core–shell microcapsules are synthesized through top‐down methods involving emulsification or ultrasonication.^[^ ^38^ ^]^ Scanning electron microscopy (SEM) images in **Figure** **3**a reveal the morphologies of the resulting microcapsules, with their size distribution analyses provided in Figure S1 (Supporting Information). The two methods generate microcapsules of distinctive sizes, as shown in Figure 3b. The size also continuously decreases by extending the process duration. Accordingly, the size modulation spans two orders of magnitude, ranging from 1.0 ± 0.4 to 151.4 ± 37.3 µm. Despite the significant variations in microcapsule size, the volume‐dependent formulation mechanism of the emulsion gel remains valid, as confirmed by systematic rheological measurements of a representative mixture containing microcapsules sized at 4.4 ± 1.6 µm (refer to Figure S17, Supporting Information). In this context, all microcapsules can be mixed with the SIS solution to form emulsion gels at high loadings, specifically ϕ = 80.8%. These emulsion gels demonstrate the suitability for 3D printing, generating a star‐shaped pattern through a 200 µm nozzle, as depicted in Figure 3c. Notably, no clogging issues are encountered during the printing process, even for microcapsules approaching the nozzle dimension. The robust printability of these emulsion gels is likely attributed to the inherent deformability of the microcapsules. However, the liquid metal tends to spread over the printed filaments using the largest microcapsules (151.4 ± 37.3 µm), indicating their undesired rupture when passing through the fine nozzle. This constraint sets a practical upper limit for the selection of liquid metal microcapsules in formulating printable inks.

Emulsion gels solidify into insulating composites, regardless of the size of the microcapsules used. Although the presence of surfactant shells and elastomers promotes the stability of liquid metal microcapsules inside the emulsion gel, these components hinder the formation of a conductive network in the microcapsule composites.^[^ ^32^, ^36^ ^]^ Mechanical activation is necessary to establish electrical conductivity, as depicted in Figure 3d. Stretching the microcapsules causes them to break, releasing the liquid metal and forming a conductive network.^[^ ^34^ ^]^ While the applied strain influences this activation process, the resulting conductivity shows only marginal improvements even at maximum strain, as depicted in Figure S18 (Supporting Information). Alternatively, the peeling process involves applying both pressing and stretching forces to the microcapsules, augmenting local stress for liquid metal release.^[^ ^47^ ^]^ SEM images in Figure 3e reveal the change in microstructure following mechanical activation. The original composite exhibits a particulate morphology consisting of isolated microcapsules. After stretching activation, the composite transforms into a continuous track with a textured surface, indicating the presence of inactivated microcapsules. In comparison, the conductive track activated by peeling displays a smooth and uniform surface, suggesting enhanced activation efficacy. As depicted in Figure 3f, the conductivities achieved by peeling surpass those attained by stretching, consistent with the observed microstructure characteristics. The activated conductivity rises as the microcapsule size increases, plateauing at ≈35.6 µm. This trend arises from the reduced stiffness of large microcapsules, making them more susceptible to mechanical damage. Notably, an impressive conductivity of 2.2 × 10^4^ S cm^−1^ has been attained, standing out among stretchable conductors.

We further investigate the impact of feature dimension on the activated conductivity. A standard microcapsule size of 108.6 µm is chosen here for these tests. The relationship between conductivity and the width of the printed features is illustrated in Figure 3g. While the conductivity remains nearly constant for wide tracks, a gradual decrease in conductivity is observed for line widths below 1 mm, indicating minor confinement effects. Figure 3h presents electrical conductivity and sheet resistance versus thickness for 3D printed features. The conductivity initially increases slightly with thickness up to 350 µm. As the thickness increases further, the conductivity starts to decrease, likely due to reduced stress transfer. It is noteworthy that the conductivity maintains a high value of 1.7 × 10^4^ S cm^−1^ even at a thickness of 1000 µm. Additionally, the sheet resistance consistently decreases with increasing thickness. Notably, the sheet resistance of 1000 µm‐thick features is 0.54 ± 0.04 mΩ sq.^−1^, comparable to 1 oz copper foils (≈34.8 µm) commonly used in commercial printed circuit boards. An advantage of 3D printing is the ability to easily create high‐height profile features by stacking multiple print layers, effectively reducing sheet resistance and ohmic loss. This fabrication capability plays a crucial role in bridging the performance gap between liquid metal‐based conductors and traditional solid metals, presenting significant potential for diverse applications.

In extrusion‐based 3D printing, the selection of nozzles plays a crucial role in determining the build speed and precision. For example, linear tracks have been created using nozzles with diameters of 200 and 600 µm, as depicted in Figure S19 (Supporting Information). These features show similar conductivity after the peeling activation. Notably, this activation step is independent of the entire printing procedure, ensuring consistent conductivity regardless of printing parameters.

### 2.4 Electromechanical Properties of Liquid Metal Conductive Features

**Figure 4:**
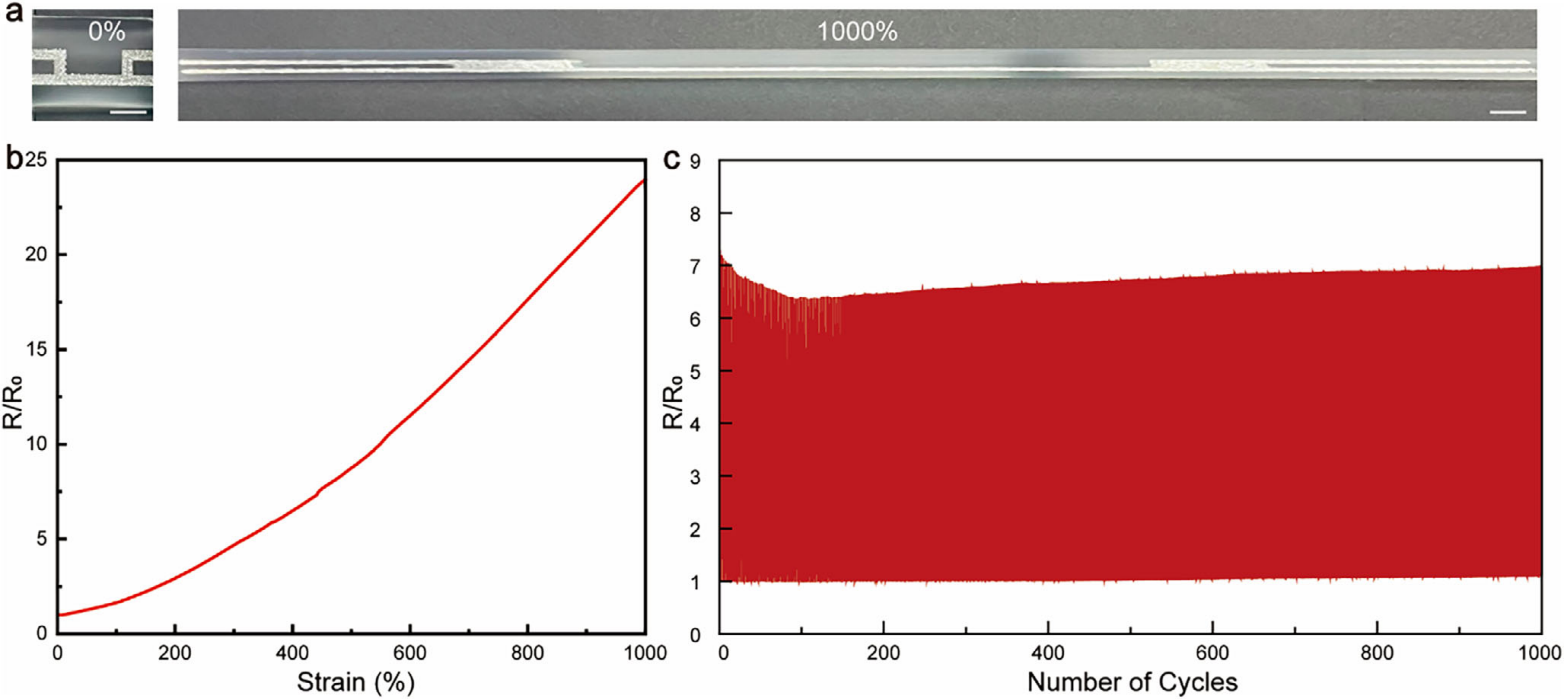
Electromechanical properties of activated liquid metal conductive features. a) Optical images showing a representative conductive feature at 0% and 1000% strains. Scale bars: 1 cm. b) Normalized resistance as a function of the uniaxial tensile strain. c) Normalized resistance during stretch cycles to 400% strain.

The activated liquid metal features are subjected to systematic tensile testing. The optical images in **Figure** **4**a reveal their impressive deformability, capable of reaching an ultrahigh strain of 1000%. Figure 4b presents the electromechanical characteristics of a representative 170 µm‐thick conductor having a sheet resistance of 30 mΩ. The resistance increases gradually with tensile strain, exhibiting normalized resistance values of 1.0, 6.2, and 22.7 at 0%, 400%, and 1000% strains, respectively. While bulk liquid metal exhibits a rapid resistance increase under strain due solely to the geometric effect of longitudinal extension and cross‐sectional contraction,^[^ ^48^ ^]^ the activated liquid metal composite shows a significantly lower relative resistance change (refer to Figures S20 and S21, Supporting Information). This difference likely stems from the formation of a 3D interconnected conductive network within the activated composite, which mitigates resistance variations.^[^ ^34^, ^49^ ^]^ Additionally, the strain‐dependent resistance is largely reversible, with overlapping loading and unloading curves, except for slight deviations at extremely high strains (see Figure S22, Supporting Information). The irreversible change in resistance after significant stretching is attributed to the residue strain in the elastomer substrate, as shown in Figure S23 (Supporting Information). To assess durability, the liquid metal feature is evaluated by 1000 stretch–relaxation cycles to 400% strain, as depicted in Figure 4c. The change in resistance remains stable throughout this fatigue test, highlighting its reliability for long‐term applications. Interestingly, the resistance at the stretched state shows a marginal decrease in the initial ten cycles, indicating that the rupture of inactive microcapsules contributes to the conductivity.

### 2.5 Stretchable LED Displays Fabricated through 3D Printing

**Figure 5:**
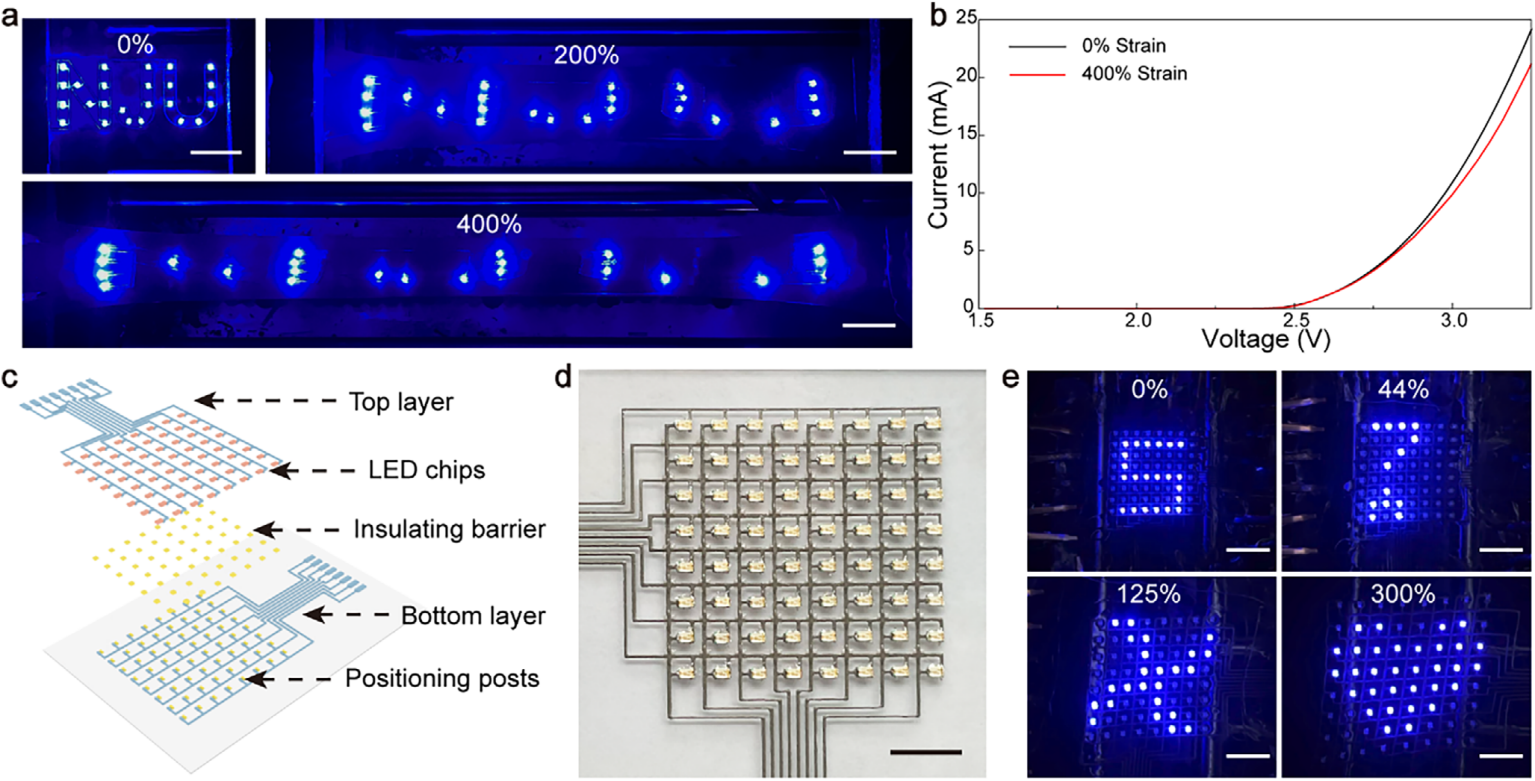
Stretchable LED displays fabricated through 3D printing. a) Optical images showing an illuminating “NJU” LED array at 0, 200%, and 400% tensile strains. Scale bars: 2 cm. b) Current−voltage curves of the LED matrix at 0 and 400% strains. c) Schematic illustrating the architecture of the stretchable LED matrix display. d) Optical images of an as‐prepared LED matrix display. Scale bars: 1 cm. e) Optical images of an LED matrix showing number and graphic patterns at 0%, 44%, 125%, and 300% area strains. Scale bars: 2 cm.

Extrusion‐based 3D printing presents an attractive fabrication platform for stretchable electronic devices. We apply this advanced technique to develop a stretchable LED display. Initially, an “NJU”‐patterned LED array comprising 24 chips is created, as schematically illustrated in Figure S24 (Supporting Information). The manufacturing process involves defining liquid metal microcapsule patterns on an SIS substrate through single‐layer prints, encompassing contact pads and electrical interconnects. These conductive features demonstrate a low sheet resistance of 2.69 mΩ sq.^−1^ after activation via peeling. LED chips are positioned on the pads and then gently pressed with a tweezer, establishing connections with the liquid metal conductor for significantly reduced contact resistance (see Figure S25, Supporting Information). As illustrated in **Figure** **5**a, the resulting LED array exhibits exceptional deformability, maintaining consistent luminous patterns upon stretching up to 400% strains. The current−voltage curve in Figure 5b displays a marginal decrease in current under substantial tensile deformations, suggesting stable light emission intensity. The highly conductive liquid metal features effectively mitigate ohmic losses, ensuring reliable operation of the LED circuit even under highly deformed conditions. Additionally, an LED matrix display is constructed based on two separate layers of circuit patterns, as schematically illustrated in Figure 5c. The liquid metal emulsion gel ink defines the conductive features, whereas the ultraviolet (UV)‐curable elastomer ink forms insulating barriers and positioning posts (Figures S26 – S28, Supporting Information). This elastomer ink has been confirmed to exhibit minimal shrinkage during cross‐linking (see Figure S29, Supporting Information). The positioning posts are used to secure the position of LED chips under significant tensile deformations. The optical image in Figure 5d presents a representative 8 × 8 LED matrix display, with individually addressable LEDs demonstrating arbitrary graphic information, such as numbers, letters, and drawings. This display exhibits compliant mechanical properties, accommodating biaxial deformations of up to 300% area strain (see Figure 5e). According to Figure S30 (Supporting Information), the associated change in drive current is minimal to ensure stable emissions. The device can dynamically refresh its graphic pattern under large applied strains, as illustrated in Movie S3 (Supporting Information). These findings highlight the potential of extrusion‐based 3D printing in creating deformable electronics, establishing an automatic manufacturing platform for assembling complex device architectures.

### 2.6 3D‐Printed Stretchable NFC Tags

**Figure 6:**
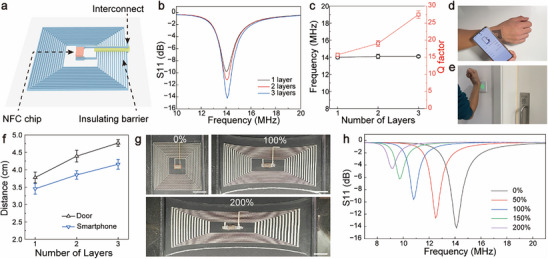
Stretchable NFC tags fabricated through 3D printing. a) Schematic showing the design of the NFC tag. b) Reflection scattering parameter (S11) versus frequency of NFC tags using conductive tracks with different layers. c) Resonant frequency and Q factor of the NFC tags versus the number of print layers. Data represents mean ± s.d. (n = 4). d) Optical image showing the operation of the NFC device with the ID information retrieved by a smartphone. e) NFC tag on the back of a hand to unlock a door. f) Maximum working distance of the NFC tag with varying numbers of print layers for door locks and smartphones. Data represents mean ± s.d. (n = 4). g) Optical images of the NFC tag at different tensile strains. Scale bars: 1 cm. h) Reflection scattering parameter (S11) versus frequency of the NFC tag at different tensile strains.

3D printing technology has been further extended to stretchable electronic devices with wireless communication capabilities. For these devices, the development of compliant and low‐resistance antennas is essential for ensuring efficient wireless communication.^[^ ^50^, ^51^ ^]^ **Figure** **6**a schematically illustrates the architecture of an NFC tag device, featuring a fifteen‐turn loop antenna and contact pads defined using liquid metal emulsion ink. Elastomer features are then printed, including an insulating barrier over the antenna and a position post between the contact pads. An NFC chip is mounted onto the contact pads and encapsulated with an elastomer layer. The optical image in Figure S31 (Supporting Information) shows an NFC tag device fabricated using this process.

A notable concern for NFC devices is the ohmic loss of the antenna, as it directly impacts radiation efficiency.^[^ ^52^ ^]^ To address this issue, the number of printed layers is adjusted to control the aspect ratio of the conductive tracks in the loop antenna. The corresponding sheet resistance is also effectively regulated, with 3.0, 1.5, and 0.9 mΩ sq.^−1^ for 1, 2, and 3 layers, respectively (Figure S32, Supporting Information). Despite the increased height of multilayer prints, the extruded elastomer still effectively covers the antenna for encapsulation purposes, ensuring the overall success of the fabrication process (see Figure S33, Supporting Information). The return loss (S11) spectra of NFC tag devices, as shown in Figure 6b, are acquired using a vector network analyzer. In Figure 6c, these devices exhibit resonant frequencies close to the standard frequency of the NFC system at 13.56 MHz. Notably, the absorbed energy increases with the layer number, resulting in boosted Q factors. The loop antenna in a trilayer print achieves a Q factor of 27.5, comparable to commercial devices.^[^ ^53^ ^]^ Thus, high‐aspect‐ratio conductive tracks significantly reduce ohmic loss in NFC devices, thereby improving wireless power efficiency. The 3D printing‐based fabrication not only streamlines the manufacturing process but also allows for tailored antenna designs that optimize performance characteristics.

These devices are readily attached to the skin, resembling tattoo‐like tags. As a smartphone approaches, the stored identity information in the tag can be wirelessly retrieved via communication protocols (Figure 6d,e). In addition, the tag device affixed to the back of a hand allows users to unlock a door by simply swinging their hands, which may find uses in access control. These practical operations of the NFC devices are demonstrated in Movie S4 (Supporting Information). As expected, the maximum working distance in these applications increases with the number of print layers (Figure 6f). Moreover, the NFC device demonstrates excellent deformability to accommodate uniaxial strains of up to 200%, as illustrated by optical images in Figure 6g. The corresponding return loss spectra in Figure 6h reveal that the resonant frequency shifted to lower values upon stretching due to an expanded loop antenna with increased inductance (see Figure S34, Supporting Information). Given the increased resistance, the stretched antenna has widened frequency responses and decreased Q factors, effectively counteracting the impact of their resonate frequency shifts. The stretched device can unlock the door at up to 150% strain despite a gradually reduced working distance (see Figure S35, Supporting Information). Furthermore, the NFC tag attached to the wrist exhibits stable operation in bent conditions, as depicted in Movie S5 (Supporting Information). These demonstrations illustrate the robust deformability and practical unity of 3D printed NFC tags in real‐world scenarios.

## 3 Conclusion

In summary, we have developed a two‐step process for generating liquid metal emulsion gels suitable for extrusion‐based 3D printing. Liquid metal microcapsules via top‐down synthesis are mixed with various polymer solutions or precursors, forming emulsion gels after surpassing the volume threshold for random close packing. This emulsion gel features shear‐thinning responses and yield flow behaviors, making it suitable for 3D printing. After solidification through solvent evaporation, the resulting composite displays minimal shrinkage of less than 4%, attributed to the densely packed microcapsules that provide excellent structural retention. Once activated, the conductive features exhibit an exceptional conductivity of 2.2× 10^4^ S cm^−1^, ultrahigh deformability of up to 1000% strain, and resilience against repetitive deformations. These properties have been leveraged to create stretchable devices with multilayered circuit patterns through 3D printing, such as LED matrix displays and skin‐attachable NFC tags. This additive manufacturing process can substantially reduce the ohmic losses of liquid metal‐based devices and circuits by conveniently stacking multiple print layers. The liquid metal emulsion gel developed here serves as a versatile ink platform for 3D‐printed stretchable electronics.

## 4 Experimental Section

### Materials

Chemical reagents and raw materials were commercially acquired, including SIS (D1164) from Kraton Corporation, thermoplastic polyurethane (TPU, AR‐62A) from Lubrizol Inc., polydimethylsiloxane (PDMS, Sylgard 184) from Dow Corning Co., PVDF‐HFP (Daiel G801) from Daikin Industries, acrylate monomer (CN9021 NS) from Sartomer Co., Ltd., silica nanoparticles (AEROSIL R974) from Evonik Degussa GmbH, 2,4,6‐trimethylbenzoyldiphenyl phosphine oxide (TPO) from Shanghai D&B Biotechnology Co., Ltd, and 11‐mercaptoundecanoic acid from Shanghai Macklin Biochemical Co., Ltd. N‐pentanol, dimethylformamide (DMF), xylene, mesitylene, and dichloromethane were all purchased from Shanghai Aladdin Biochemical Technology Co., Ltd. Silica nanoparticles had been thoroughly dried in a vacuum oven before use. The liquid metal was formed by alloying Ga (99.99%), In (99.99%), and Sn (99.999%) at 75 °C for 2 h inside a nitrogen glovebox, according to a weight ratio of 68.5: 21.5: 10.

### Preparation of Printable Liquid Metal Emulsion Gels

The top‐down synthesis of liquid metal microcapsule involved carefully transferring 10 grams of liquid metal into a 10 mL mixture of 0.25 m 11‐mercaptoundecanoic acid in N‐pentanol. This mixture was processed using either a FJ‐200 high‐speed homogenizer from Changzhou Guoyu Instrument Manufacturing Co., Ltd. at a speed of 11 000 rpm or a 900‐W probe sonicator (ATPIO‐900D, Nanjing Xianou Instrument Manufacturing Co., Ltd.) at a 10% power setting. The duration was adjusted to regulate the size of the resulting microcapsules (refer to Figure S1, Supporting Information). Following the formation of microcapsules, they were thoroughly washed with dichloromethane. Liquid metal microcapsules and the polymer solution/precursor were thoroughly homogenized at 2000 rpm for 3 min using a planetary mixer (JF‐RVITV‐150, Shenzhen Junfeng Technology Co., Ltd.). Predominately SIS solution was used (20 w/v % in xylene) in this study. Other options involve PVDF‐HFP solution (20 w/v % in DMF), TPU solution (20 w/v % in DMF), and PDMS (base: curing agent = 10: 1).

### Preparation of Printable UV Curable Elastomer Inks

The elastomer precursor was formulated by adding 1 w/w% TPO photoinitiator to the acrylate monomer. Next, silica nanoparticles were incorporated into the elastomer precursor at 10 w/v % as a rheological modifier. The mixture was homogenized in the planetary mixer at 2000 rpm for 5 min.

### 3D Printing Process

Unless stated otherwise, an optimized formulation was primarily used, involving 108 µm‐sized liquid metal microcapsules and SIS binder in a volumetric concentration of 80.8%. Inks were filled into 10 mL syringe barrels that were equipped with a Musashi dispensing needle and mounted onto a motorized computer‐controlled three‐axis stage (± 0.01 mm, Shenzhen Kingwonda Electromechanical Co., Ltd.). Ink extrusion was controlled with AD3000C pneumatic regulators from Shenzhen Haiyu Electromechanical Equipment Co., Ltd. All printing paths were generated by converting the feature design into compatible G‐codes using Logoshop Designer software. Optimized printing conditions involved 10 mm s^−1^ speed and 100 kPa pressure for the liquid metal emulsion gel and 3 mm s^−1^ and 400 kPa for the elastomer ink. As‐printed features of liquid metal emulsion gel were dried into solid composites by raising the sample stage temperature to 120 °C for 30 min. The elastomer ink is cured with 365 nm irradiation at 100 mW cm^−2^ for 10 s by a UVGO LED curing lamp from Zhongshan Yimiao Optoelectronic Technology Co., Ltd.

### Mechanical Activation

All printed liquid metal composites underwent mechanical treatments to establish their electrical conductivity. For the stretching activation, a tensile strain of 100% was applied to the samples using a homemade uniaxial motorized linear translational stage. For the peeling activation, samples were carefully separated from the glass substrate at a controlled peeling angle of 150°. These mechanical manipulations led to a transition of the insulating composites into highly conductive states.

### Material Characterizations

Optical microscopy and optical topographic images were acquired using a Keyence VK‐X1000 laser scanning microscope. Optical images were captured with a Fujifilm X‐T10 digital camera. Scanning electron microscopy (SEM) images were taken with a Zeiss Gemini 500 field emission scanning electron microscope. Uniaxial tensile stress‐strain curves were obtained using a Shimadzu AGS‐X universal testing machine equipped with a 50 N load cell, operating at an extension rate of 10 mm min^−1^. Rheological measurements were conducted with a HAAKE RheoStress 600 rheometer utilizing 35 mm‐diameter parallel plates with a fixed gap of 0.5 mm. The viscosity was measured across a shear rate range from 0.1 to 200 s^−1^, and dynamic oscillatory shear measurements were performed at a fixed frequency of 1 Hz. Electrical resistance was measured using a Gwinstek GOM‐805 milliohm meter in a four‐probe configuration. Tensile strains were applied using homemade uniaxial/biaxial motorized linear translational stages. The resistance was normalized to its value at the relaxed state to illustrate relative changes during stretching. Conductive features of the bulk liquid metal on elastomer substrates were fabricated using a template‐based technique.^[^ ^19^, ^54^ ^]^ Briefly, a Cr/Cu (20 nm / 100 nm) layer was thermally evaporated through shadow masks to create a four‐probe measurement pattern. In the presence of hydrochloric acid solution (4%, w/v), non‐oxidized liquid metal spread onto this pattern, forming the desired features due to selective wettability.

### Fabrication and Evaluation for Stretchable LED Arrays

Conductive patterns, including electrical interconnects and contact pads, were defined with the liquid metal microcapsule gel on an SIS substrate. The elastomer features at the crosspoint were then printed to provide electrical insulation, followed by curing with UV irradiation for 10 s. A second layer of conductive features was generated. Elastomer posts were printed beneath the chips to hold their positions. LED chips (LTW‐170TK, 0805, Lite‐On Inc.) were mounted on the contact pads and manually pressed with a tweezer. The procedure ensured that the leads made contact with the sintered liquid metal for reliable electrical connections.^[^ ^55^ ^]^ The elastomer posts were cured through UV light irradiation. A layer of SIS was spray deposited over the entire sample for encapsulation using an ultrasonic spray system from Hangzhou Dowell Ultrasonic Technology, Co., Ltd. The positioning posts and this encapsulation layer collectively not only hold the chips in place but also prevent uncontrolled liquid metal flow and leakage. A copper‐clad polyimide‐based flexible connector was attached to the liquid metal leads for interfacing with an Arduino Uno microcontroller. A customized program installed on the microcontroller regulated the luminous pattern of the LED matrix display. Electrical characterization of the interconnected LEDs was performed using a Keithley 2634B sourcemeter, with direct contact established between the testing probes and the copper leads of the flexible connector.

### Fabrication and Evaluation for Stretchable NFC Tags

A loop coil was designed using eDesignSuite software (STMicroelectronics) to match with the selected chip for resonance at ≈13.56 MHz. The coil featured a fifteen‐turn, square‐shaped pattern measuring a 35 mm × 35 mm dimension, a 0.4 mm line width, and 0.4 mm spacing. This coil pattern was defined with the liquid metal microcapsule gel on an SIS substrate, followed by thoroughly drying at 120 °C. An elastomer bridge was printed over the coil, followed by curing with UV irradiation for 10 s. An electrical interconnect was subsequently printed over the insulating layer to join the coil and the solder pad. An elastomer post was positioned between the contact pads and then cured through UV light irradiation. A unique identifier (UID) chip (FM11RF08) was gently placed on the contact pads and manually pressed with a tweezer. Finally, a layer of SIS was ultrasonic spray coated as the encapsulant to complete the fabrication process. The electromagnetic characterization was performed using a Vector Network Analyzer (NA7632A, Deviser Electronics Instrument Co., Ltd.) equipped with a homemade loop probe. The NFC tag was placed at the center of the probe with a vertical distance of ≈2 mm. In practical applications, an NFC tag was placed within 30 mm of an NFC‐enable smartphone (Xiaomi 12). The smartphone with the NFC Tool application established communication and retrieved the information from the chip. In addition, an RFID Reader was used to write the identity information to an NFC tag, granting it permission to open the door.

## Conflict of Interest

The authors declare no conflict of interest.

## Supporting information



Supporting Information

Supplemental Video 1

Supplemental Video 2

Supplemental Video 3

Supplemental Video 4

Supplemental Video 5

## Data Availability

The data that support the findings of this study are available in the supplementary material of this article.
