# Supplementary material for: Highly Conductive Liquid Metal Emulsion Gels for Three‐Dimensionally Printed Stretchable Electronics
Source: Adv Sci (Weinh). 2025 Jul 6;12(36):e03449. doi: 10.1002/advs.202503449 (PMC12462967; doi:10.1002/advs.202503449)
Supplement: Supplementary file 1 — Supporting Information [file ADVS-12-e03449-s005.docx]

Supporting Information

**Highly conductive liquid metal emulsion gels for three-dimensionally printed stretchable electronics**

*Qianying Lu, Ting Fang, Chenyang Ye, Yanyan Li, Ming Wu, Yuping Sun, Desheng Kong^*^, Xiaoliang Wang^*^ and Yan-qing Lu^*^*


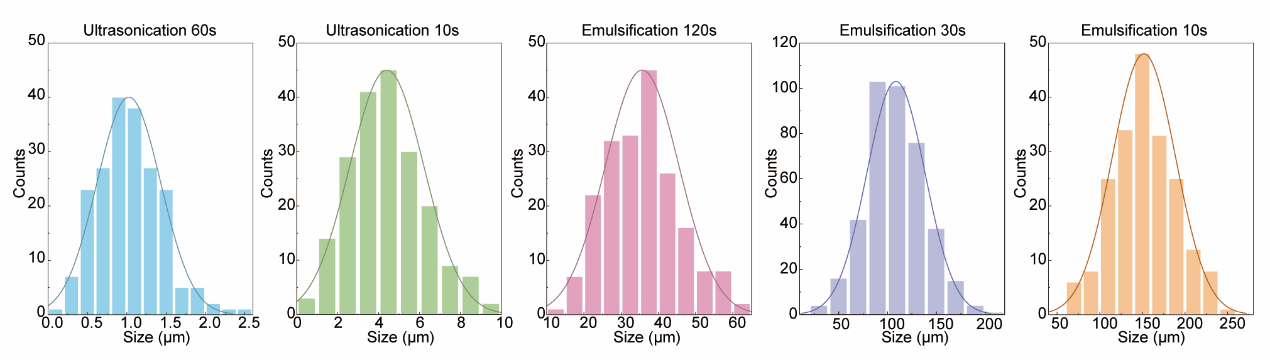


**Figure S1**. Histogram of liquid metal microcapsule size by different synthesis conditions, including ultrasonication for 60 and 10 s and emulsification for 120, 30, and 10 s. Gaussian fits of the distributions yield their characteristic sizes (from left to right) of 1.0 ± 0.4, 4.4 ± 1.6, 35.6 ± 9.9, 108.6 ± 29.8, and 151.4 ± 37.3 μm, respectively. The results indicate that ultrasonication is more effective than emulsification in reducing particle size. Additionally, the particle size decreases with increasing processing time.


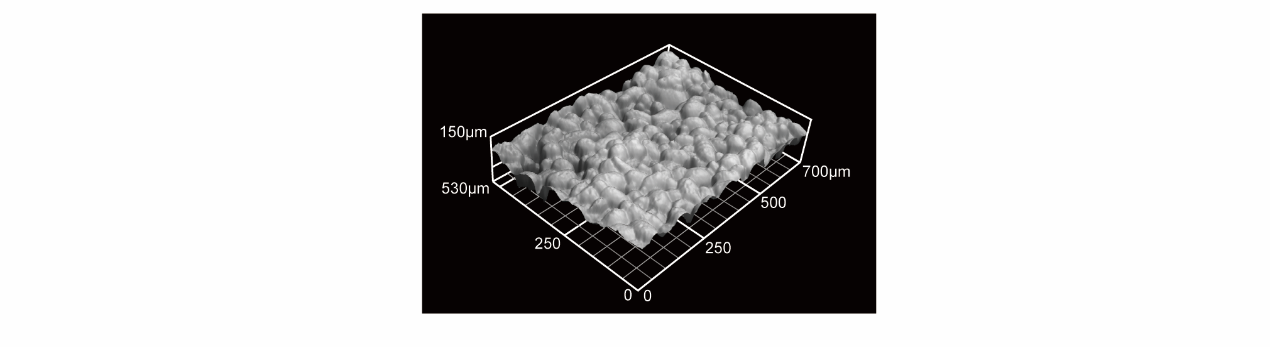


**Figure S2.** Surface topographic image of liquid metal microcapsules encapsulated within an emulsion gel. These microcapsules exhibit a close packing arrangement within the emulsion gel, contributing to its distinctive rheological characteristics.


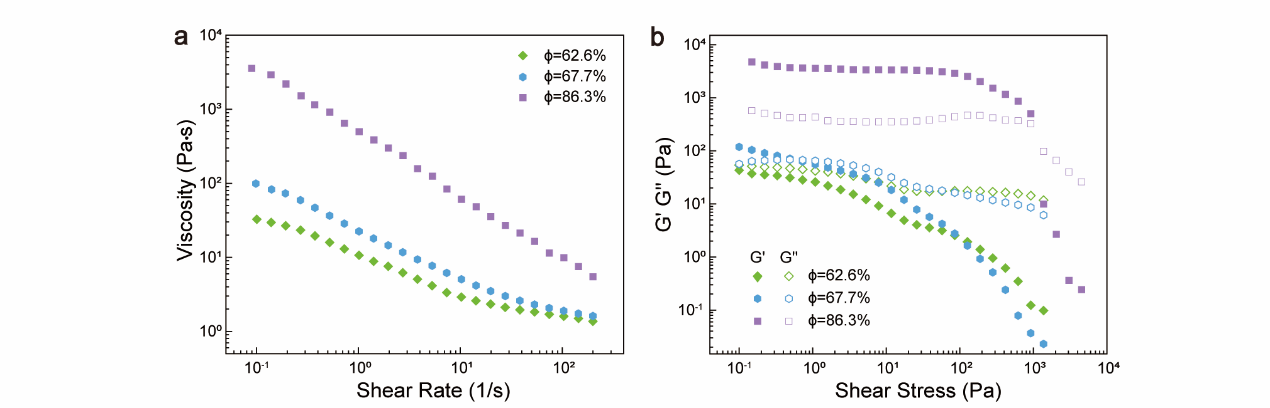


**Figure S3.** Rheological characterization on the liquid metal microcapsule and SIS solution mixtures. (a) Viscosity *versus* shear rate for microcapsule dispersion with varying volumetric concentrations. (b) Storage modulus (G′) and loss modulus (G″) as a function of shear stress. The viscosity increases with the volume fraction of liquid metal microcapsules (). While the mixture remains in a liquid state for = 62.6%, it becomes a yield-stress fluid when the volume fraction exceeds 67.7%.


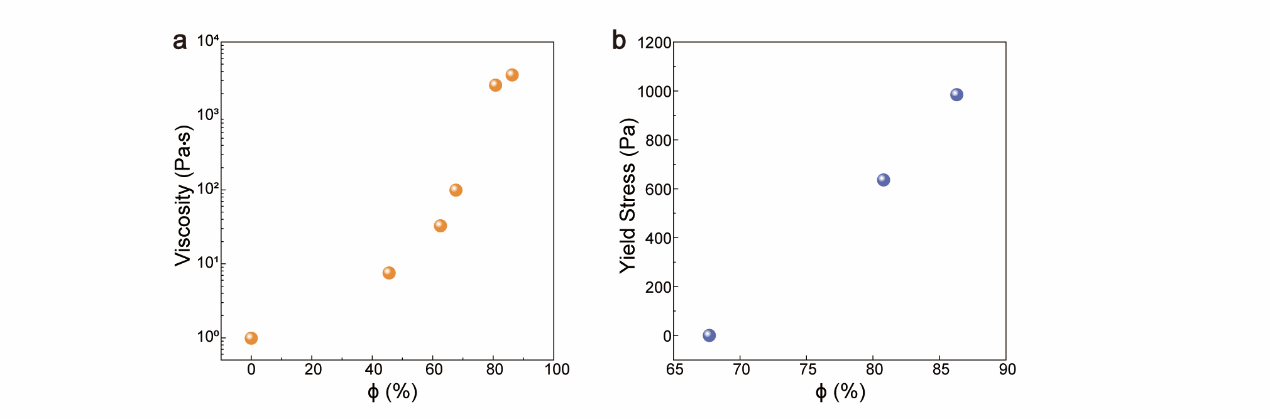


**Figure S4.** (a) Viscosity (at 0.1 s^-1^ shear rate) as a function of liquid metal microcapsule volume fraction () in its mixture with the SIS solution. (b) Yield stress *versus* liquid metal microcapsule volume fraction. Both viscosity and yield stress increase with the volume fraction of liquid metal microcapsules. It is important to note that the mixture does not form an emulsion gel when is below 67.7%.


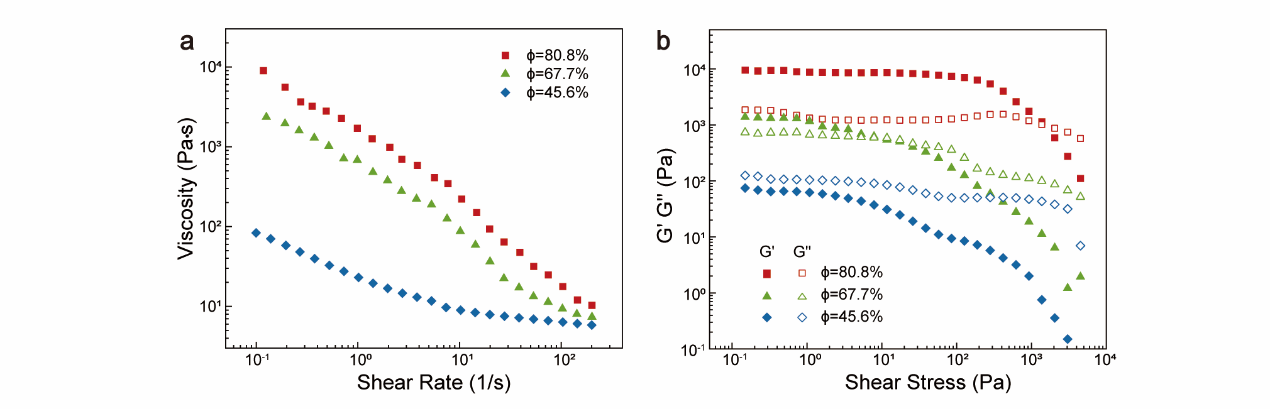


**Figure S5.** Rheological characterization on the liquid metal microcapsule with oxide shell and SIS solution mixtures. (a) Viscosity *versus* shear rate for microcapsule dispersion with varying volumetric concentrations. (b) Storage modulus (G′) and loss modulus (G″) as a function of shear stress. The viscosity increases with the volume fraction of liquid metal microcapsules (). The mixture with exceeds 67.7% exhibits yield-stress fluid behaviors.


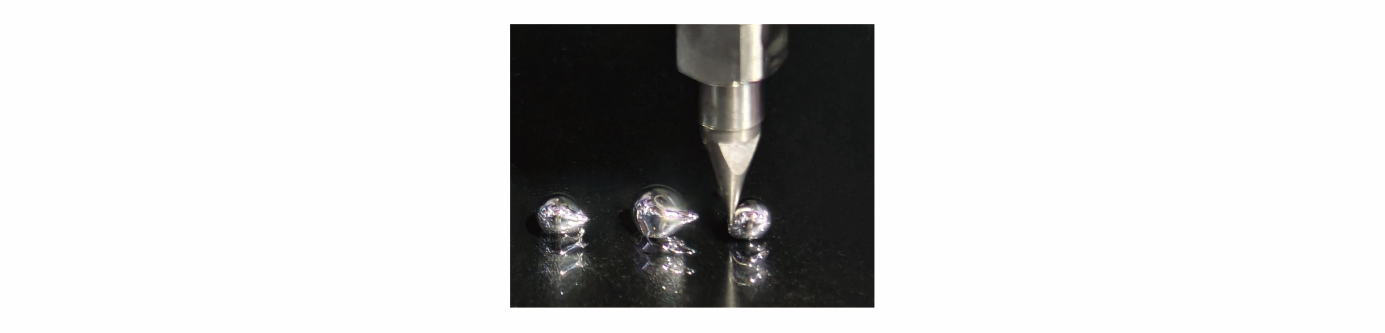


**Figure S6.** Optical image of the printing process using pure liquid metal microcapsules. During the extrusion process, the microcapsules merge into larger liquid metal droplets. This coalescence prevents the achievement of controlled printing for the desired features.


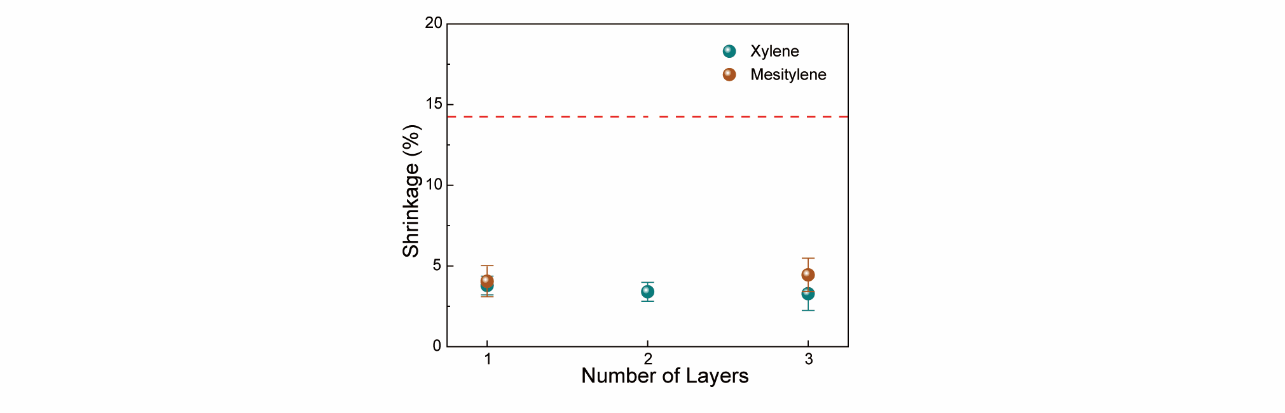


**Figure S7**. Shrinkage rate of fully dried features as a function of print layer number based on emulsion gel inks containing different solvents. The shrinkage rate of xylene and mesitylene-based emulsion gel inks are below 5%, regardless of the number of print layers. The shrinkage rate of xylene-based emulsion gel inks with single-layer, two-layer and three-layer are3.8 ± 0.6, 3.4 ± 0.6 and 3.3 ± 1.0, respectively. The shrinkage rate of mesitylene -based emulsion gel inks with single-layer and three-layer are 3.9 ± 0.8 and 4.9 ± 0.9. The dashed line represents the theoretical uniform shrinkage value of 14.3 %. Data represents mean ± s.d. (n = 4).


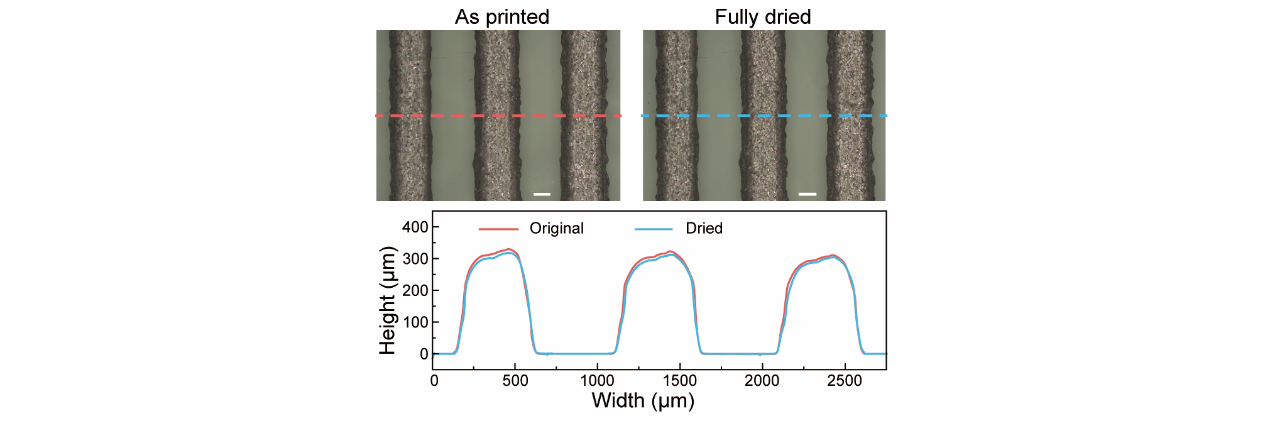


**Figure S8.** Optical microscopy images (top) and corresponding height profiles of the dashed linecuts (bottom) displaying two-layer stripe-shaped features using xylene-based emulsion gel inks in their original and thoroughly dried states. The shrinkage of two-layer features is determined as 3.4%. Scale bars: 200 μm.


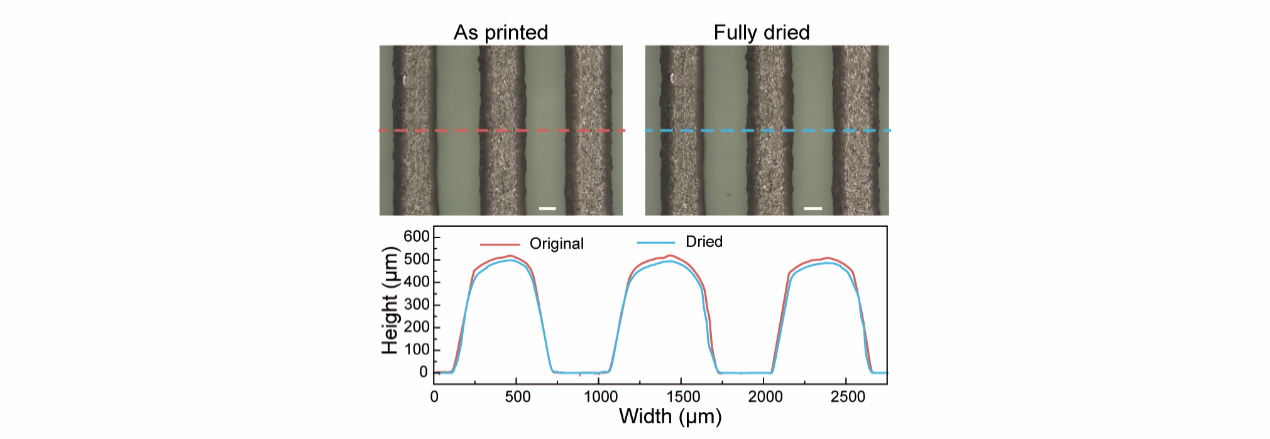


**Figure S9.** Optical microscopy images (top) and corresponding height profiles of the dashed linecuts (bottom) displaying three-layer stripe-shaped features in their original and thoroughly dried states. The shrinkage of three-layer features is determined as 3.3%. Scale bars: 200 μm.


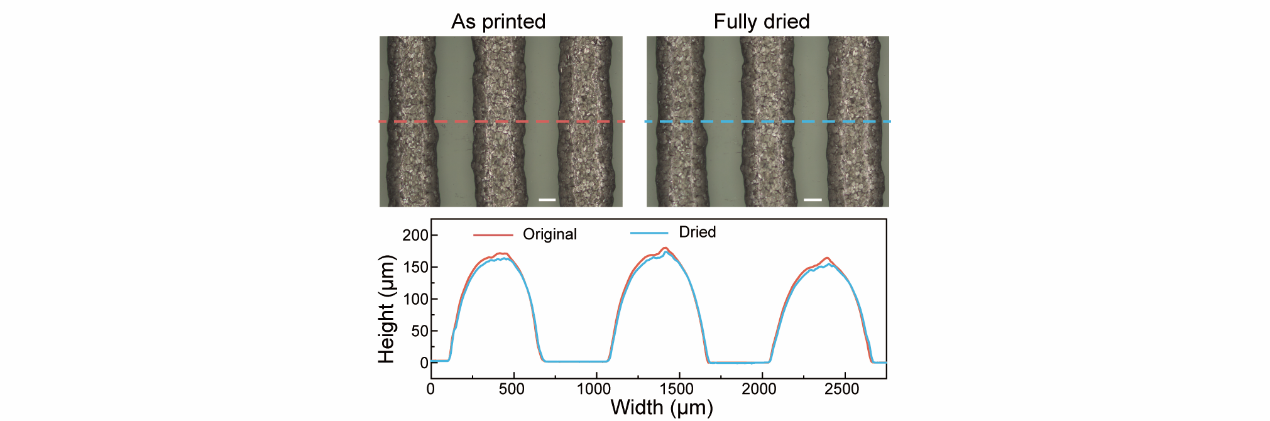


**Figure S10.** Optical microscopy images (top) and corresponding height profiles (dashed linecuts, bottom) of single-layer stripe patterns using mesitylene-based inks in the as-printed and thoroughly dried states. The shrinkage of single-layer features was about 4.0%. Scale bars: 200 μm.


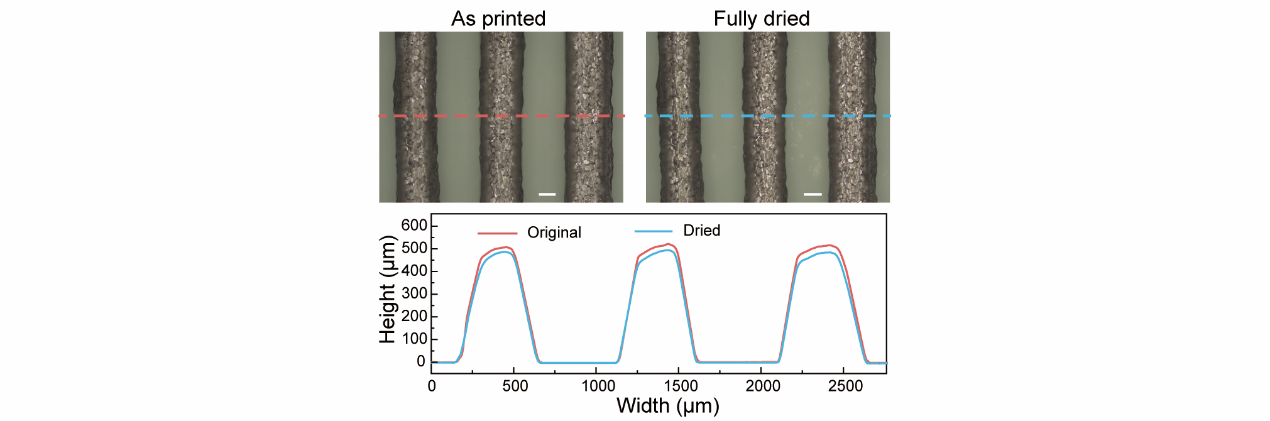


**Figure S11.** Optical microscopy images (top) and corresponding height profiles (dashed linecuts, bottom) of three-layer stripe features using mesitylene-based inks in the as-printed and thoroughly dried states. The shrinkage of three-layer features is determined as 4.9%. Scale bars: 200 μm.


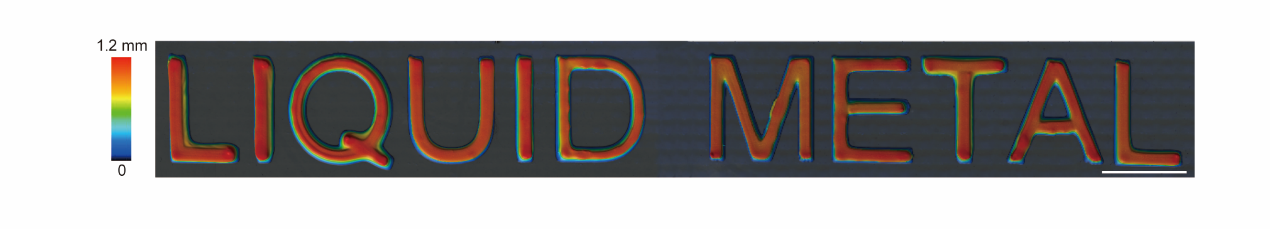


**Figure S12**. Surface topographic image of a “LIQUID METAL” feature formed by stacking multiple print layers. Multi-layer stacking enables straightforward fabrication of high-aspect-ratio structures. Scale bar: 1 cm.


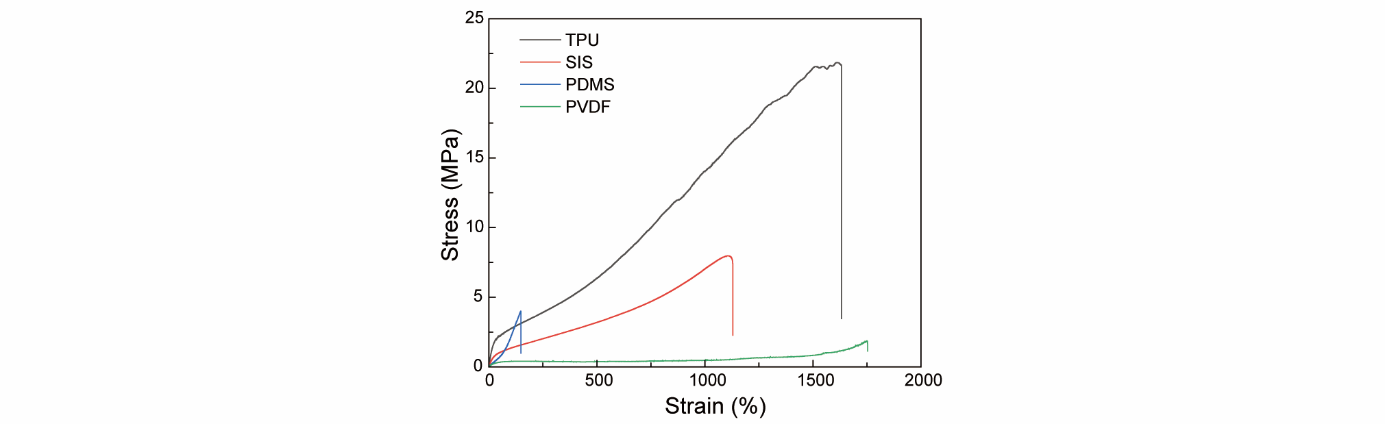


**Figure S13**. Uniaxial tensile stress–strain curves for different elastomers. Young's modulus is determined as 9.7 MPa for TPU, 4.0 MPa for SIS, 1.6 MPa for PDMS, and 1.3 MPa for PVDF-HFP, demonstrating distinct mechanical properties across materials.


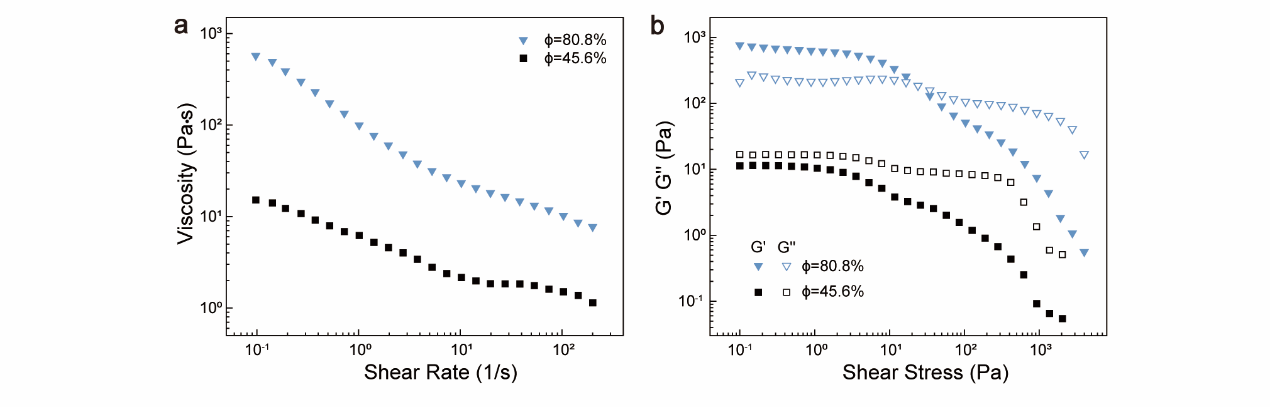


**Figure S14**. Rheological properties of liquid metal microcapsule-TPU solution mixtures. (a) Viscosity *versus* shear rate for mixtures with different microcapsule volume concentrations (). (b) Corresponding storage modulus (G′) and loss modulus (G″) as a function of shear stress. The viscosity increases with the volume fraction of liquid metal microcapsules. The mixture at = 80.8% exhibits yield-stress fluid behaviors.


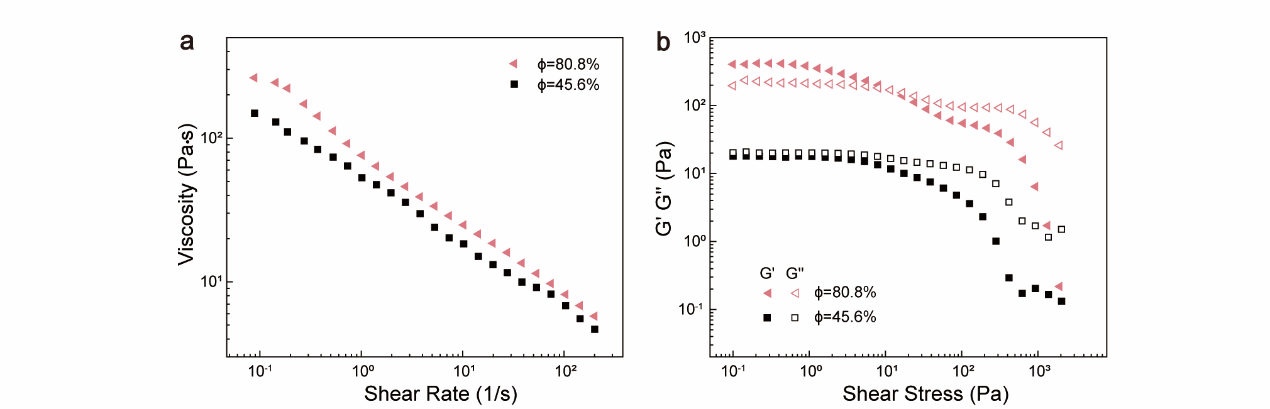


**F****igure S15.** Rheological properties of liquid metal microcapsule-PVDF-HFP solution mixtures. (a) Viscosity *versus* shear rate for mixtures with different microcapsule concentrations (). (b) Corresponding storage modulus (G′) and loss modulus (G″) as a function of shear stress. The viscosity increases with the volume fraction of liquid metal microcapsules. The mixture at = 80.8% exhibits yield-stress fluid behaviors.


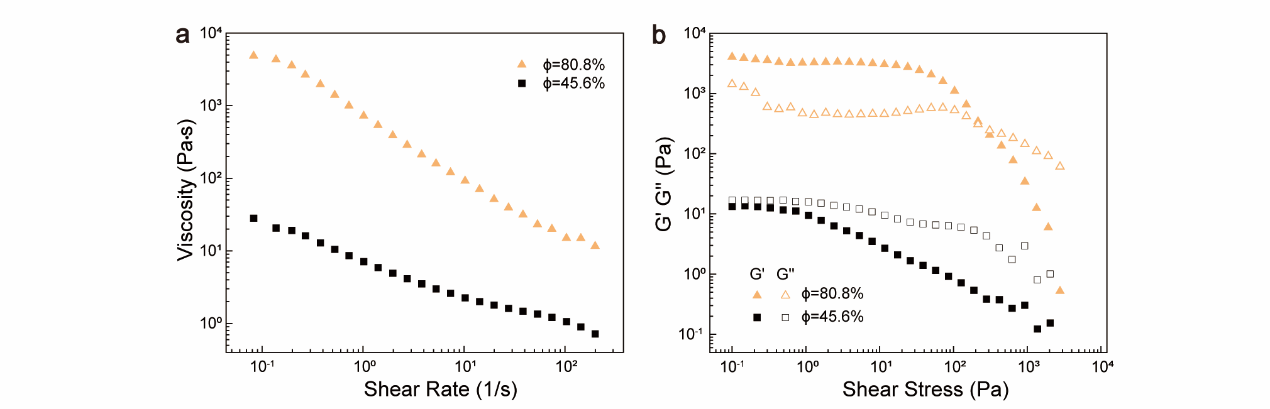


**Figure S16**. Rheological properties of liquid metal microcapsule-PDMS precursor mixtures. (a) Viscosity *versus* shear rate for mixtures with different microcapsule concentrations (). (b) Corresponding storage modulus (G′) and loss modulus (G″) as a function of shear stress. The viscosity increases with the volume fraction of liquid metal microcapsules. The mixture at  = 80.8% exhibits yield-stress fluid behaviors.


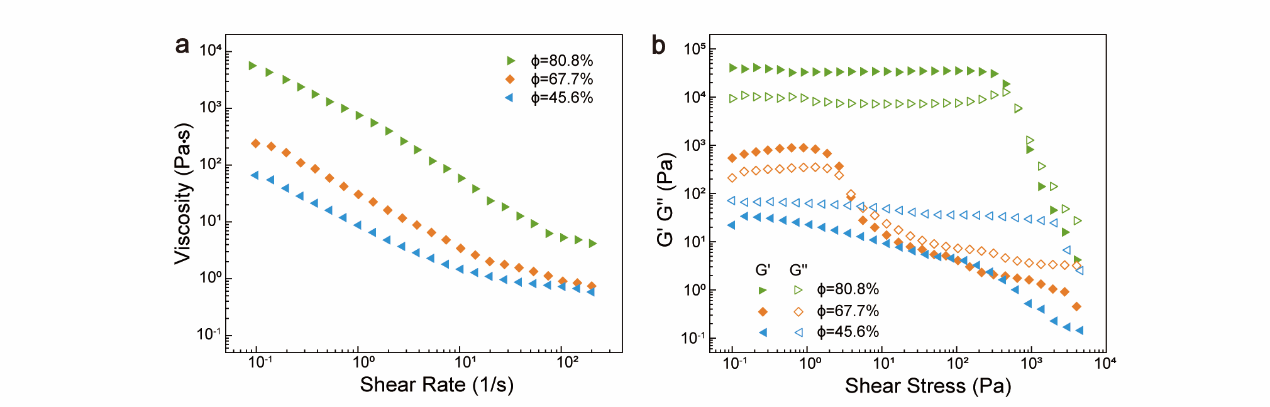


**Figure S17.** Rheological properties of fine liquid metal microcapsule dispersed in SIS solution. (a) Viscosity as a function of shear rate. (b) Storage modulus (G′) and loss modulus (G″) versus shear stress. The liquid metal microcapsules utilized have a characteristic dimension of 4.4 ± 1.6 μm. The viscosity of the mixture increases with the volume fraction of liquid metal microcapsules (). The mixture remains in a liquid state at = 45.6%. However, it transitions to a yield stress fluid at = 67.7% and 80.8%. Notably, the critical volume threshold for this transition closely aligns with the values observed for larger microcapsules.


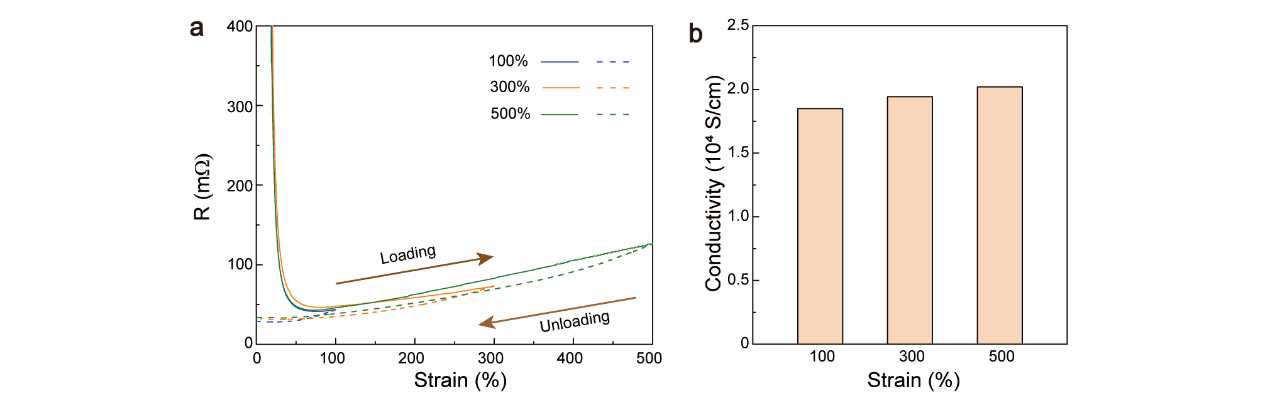


**Figure S18.** Strain-dependent stretching activation of liquid metal composites. (a) Electrical resistance evolution under applied tensile strain. (b) Achieved electrical conductivity after stretching activation with varying maximal strains.


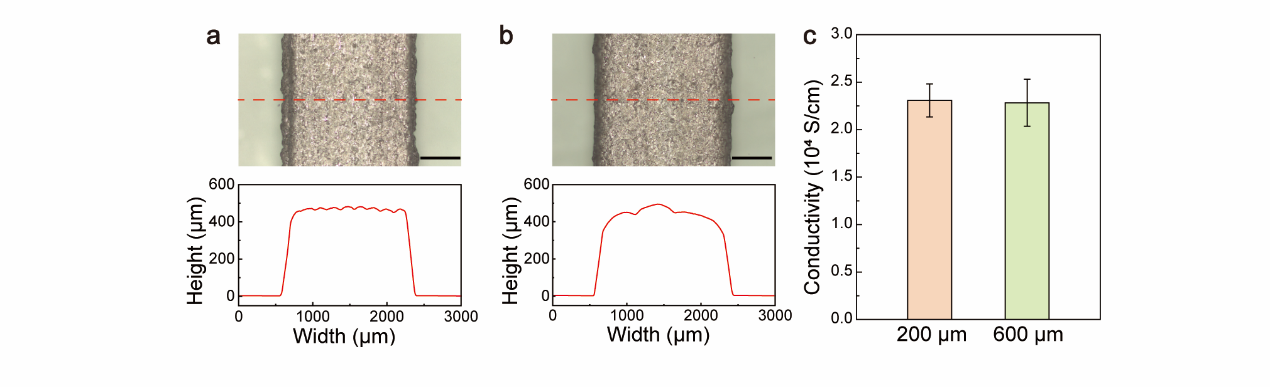


**Figure S19.** (a, b) Optical microscope images and corresponding height profiles of dashed line cuts displaying line-shaped features printed using 200 (a) and 600 (b) μm nozzles. The two features have similar appearances, regardless of the nozzle size used for printing. Scale bars: 500 μm. (c) Conductivity of the composite features activated by peeling. The conductivities of the features printed with both nozzle sizes are also quite similar. Data represents mean ± s.d. (n = 4).


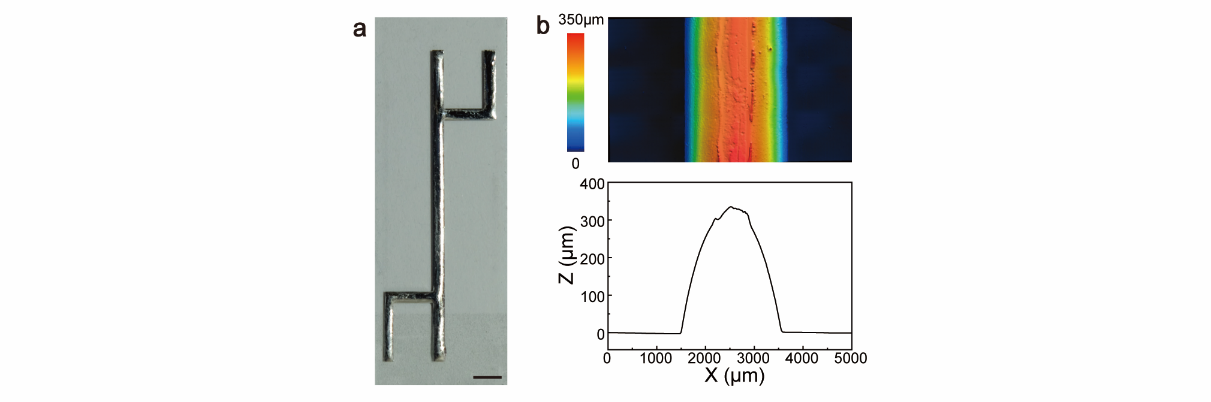


**Figure S20.** (a) Optical microscopy image showing a representative feature of bulk liquid metal for four-probe resistance measurements. (b) Surface topographic image (up) and corresponding height profile (bottom) of the liquid metal feature. This feature has an arch-shaped cross-section measuring 2050 µm in width and 334 µm in height, with a substantial dimension to ensure its bulk-type behavior.


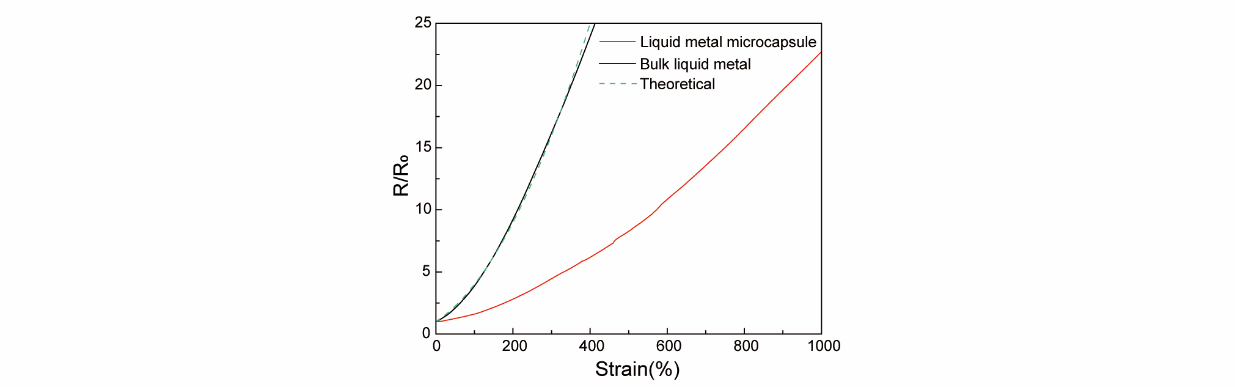


**Figure S21.** Normalized resistance (*R*/*R*_0_) versus uniaxial tensile strain for bulk liquid metals and activated liquid metal composites. The data of bulk liquid metal align with the theoretical prediction for ideal incompressible conductors, expressed as *R*/*R*_0_ = (1 + ε)^2^, where *ε* represents the uniaxial strain.^[48]^ In contrast, the resistance variations of the activated liquid metal composite are significantly smaller than that of the liquid metal, likely due to its 3D interconnected conductive networks.^[34, 49]^


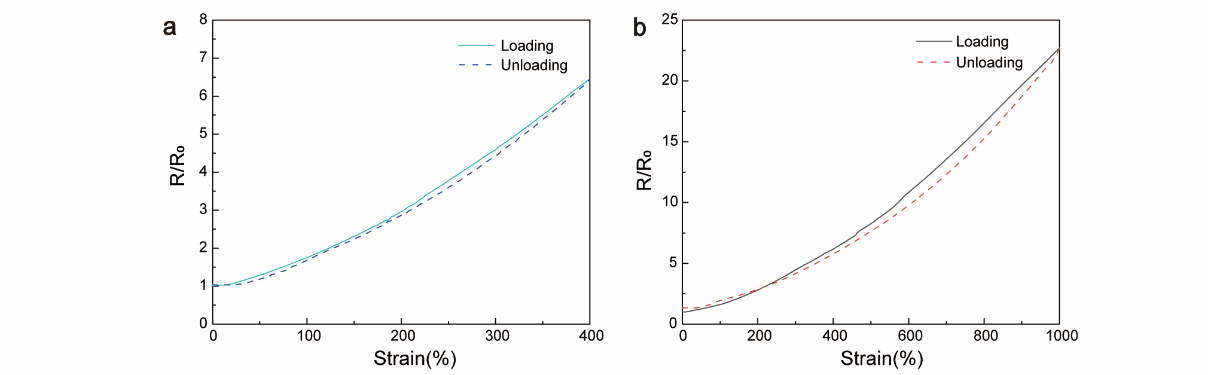


**Figure S22.** Normalized resistance (*R*/*R*_0_) of activated liquid metal composite features during a uniaxial stretching cycle, with a maximum strain of 400% (a) and 1000% (b). Due to the residual plastic strain, the irreversible change in resistance is ~4% after a 400% strain cycle and ~33% after a 1000% strain stretching cycle.


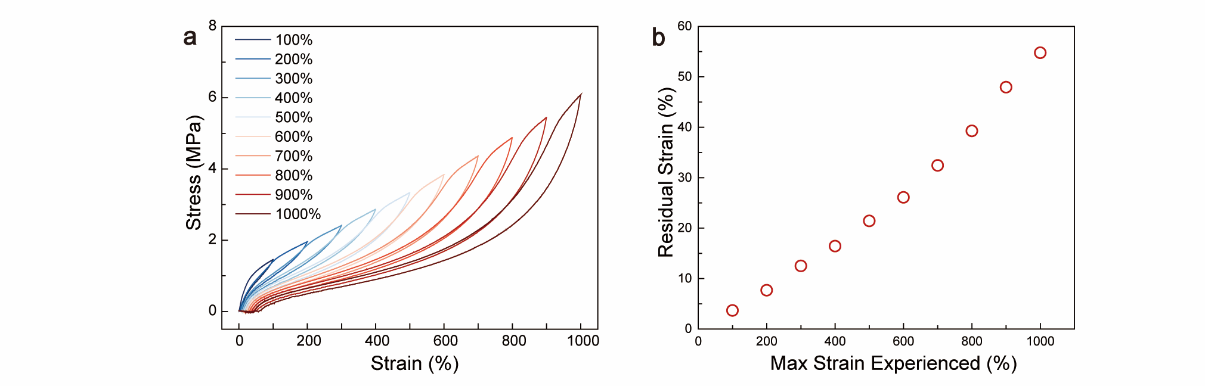


**Figure S23.** Mechanical characterization of SIS substrates. (a) Loading-unloading stress-strain curves with different maximal strains. (b) Residual strain after tensile deformation. The residual strain reflects the irreversible elongation of SIS substrate after experiencing tensile deformations.


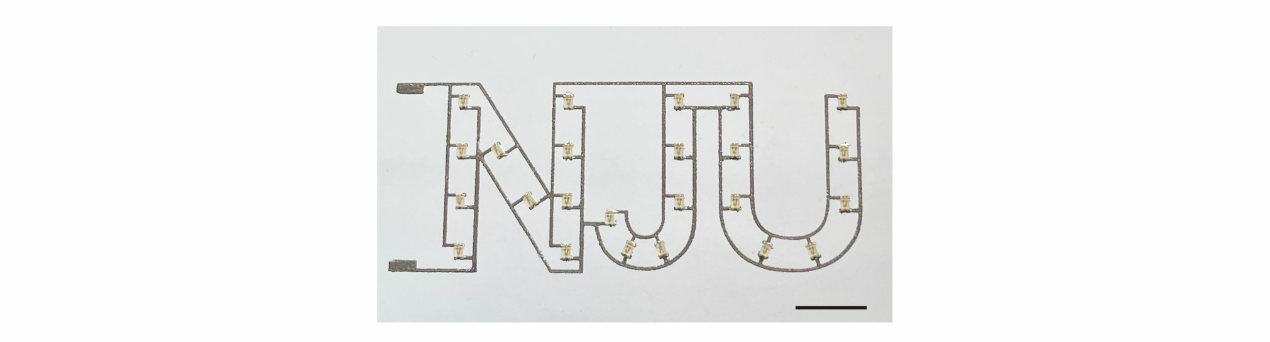


**Figure S24.** Optical image of a “NJU” LED array. Scale bar: 1 cm.


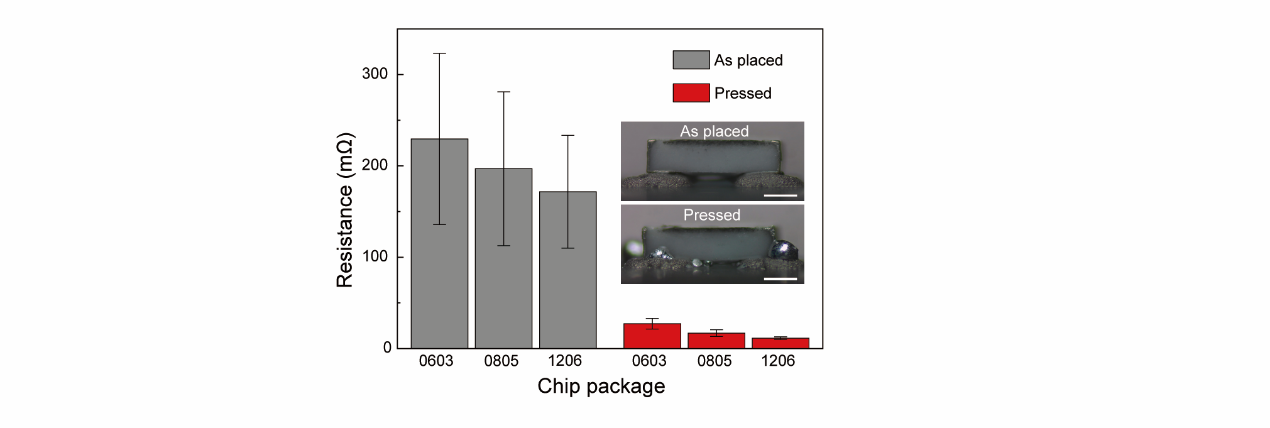


**Figure S25.** Contact resistance of activated conductive features to the chips in different package sizes. When pressed with tweezers, LED chips establish connections with the liquid metal conductor, resulting in significantly reduced contact resistance. Data represents mean ± s.d. (n = 4). Insets: Side-view optical microscope images revealing the as-placed (top) and mechanically pressed (bottom) chips on the contact pads. Scale bars: 500 μm.


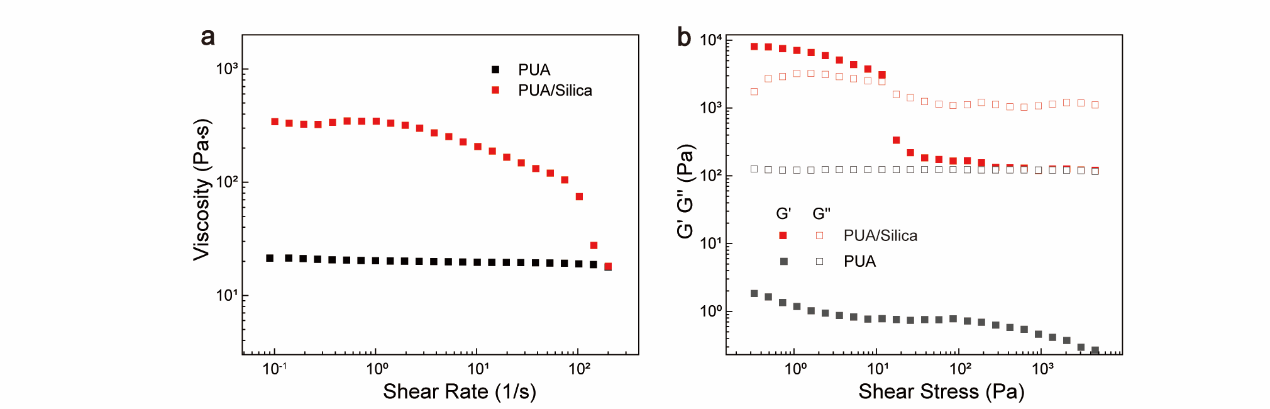


**Figure S26.** Rheological properties of the polyurethane acrylate (PUA) precursor and its formulated elastomer inks. (a) Viscosity *versus* shear rate for the PUA and PUA/silica nanocomposite. (b) Storage modulus (G′) and loss modulus (G″) as a function of shear stress. The elastomer ink is formulated by adding 10 w/w% silica nanoparticles into the PUA precursor. The addition of silica nanoparticles significantly increases the viscosity, transforming the mixture into a yield-stress fluid.


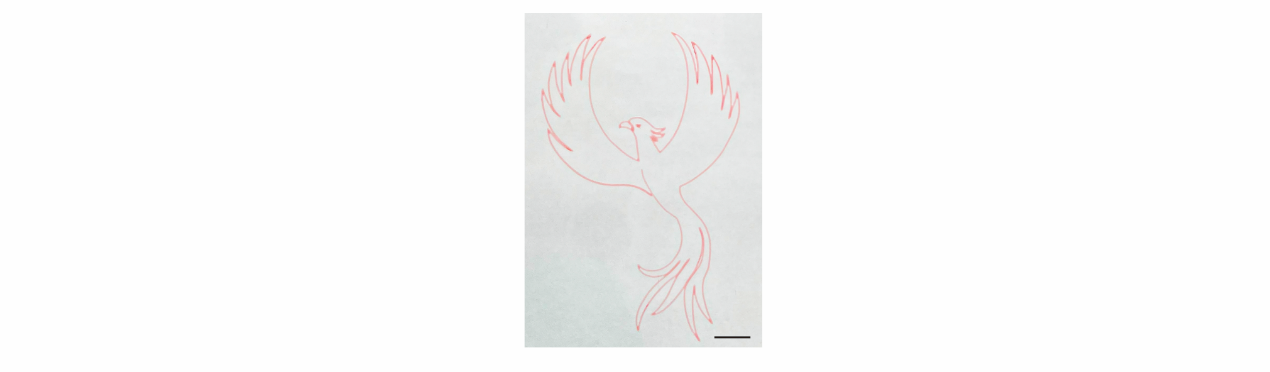


**Figure S27.** Optical image of a phoenix-shaped pattern printed using the elastomer ink. These results demonstrate the ink's capability to create high-fidelity features. Scale bar: 1 cm.


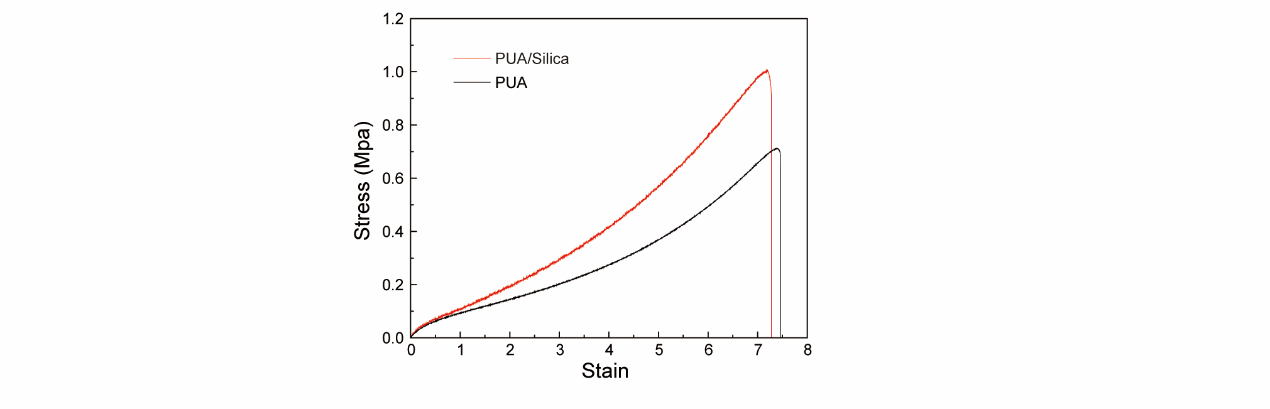


**Figure S28.** Uniaxial tensile stress-strain curves of PUA and PUA/silica nanocomposite. The measured Young's moduli are 0.2 MPa for PUA and 0.26 MPa for PUA/silica nanocomposite, respectively.


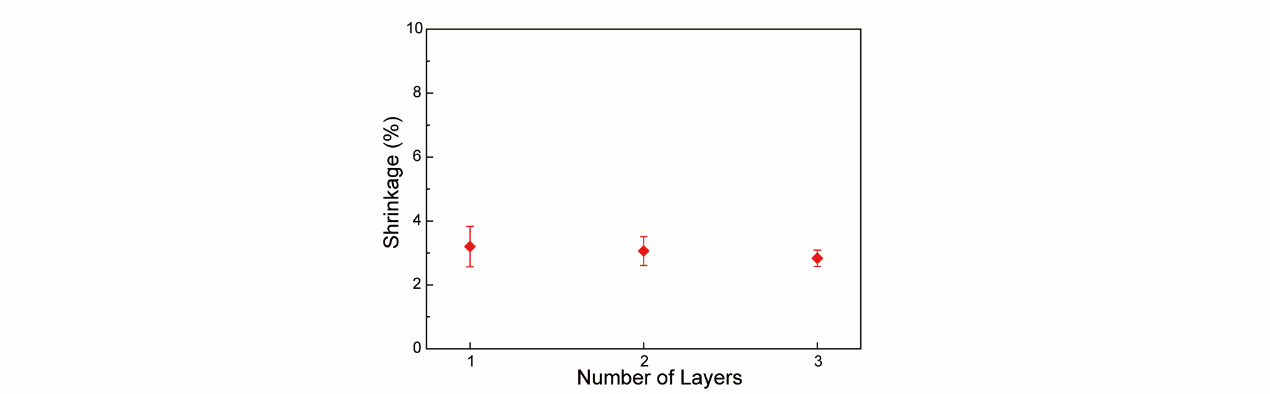


**Figure S29.** Shrinkage rate of printed elastomer features during UV crosslinking. Data represent mean ± s.d. (n = 3). These features exhibit a consistent shrinkage rate of ~3% across varying numbers of print layers. The shrinkage rate of elastomer features with single-layer, two-layer and three-layer are3.2 ± 0.6, 3.1 ± 0.4 and 2.8 ± 0.2, respectively. This minimal shrinkage aligns with the dimensional stability commonly seen in crosslinked elastomers.


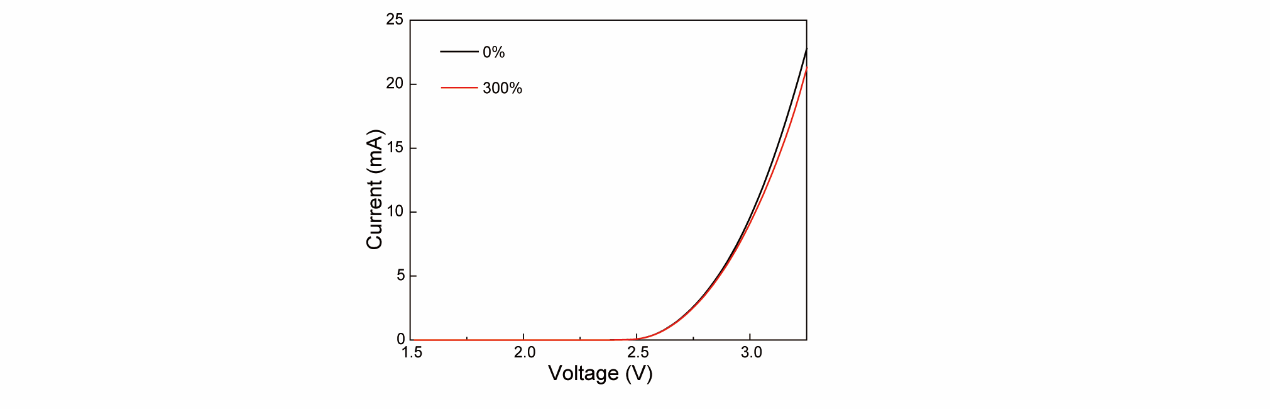


**Figure S30.** Current-voltage curves of an LED matrix display at 0% and 300% area strains, showing minimal reduction in current during stretching. This observation correlates with the slight changes in circuit resistance, which results in only minor increases in ohmic losses.


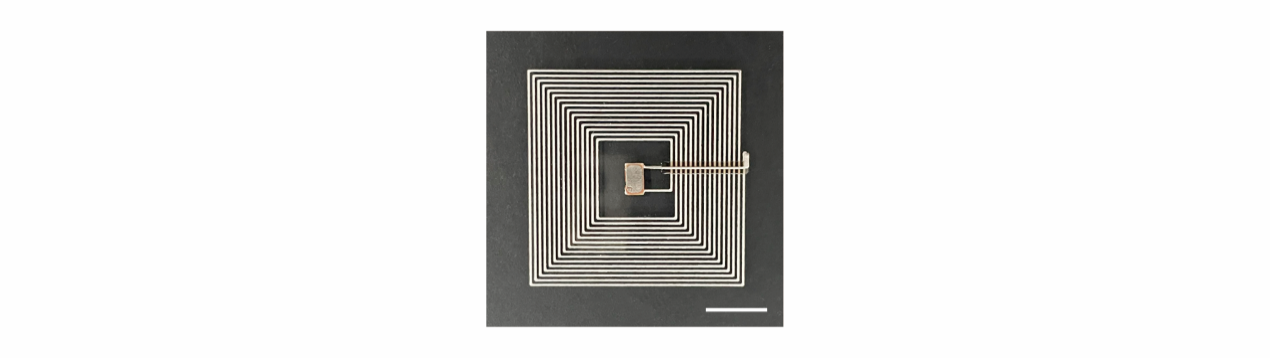


**Figure S31.** Optical image of a representative stretchable NFC tag device. Scale bar: 1 cm.


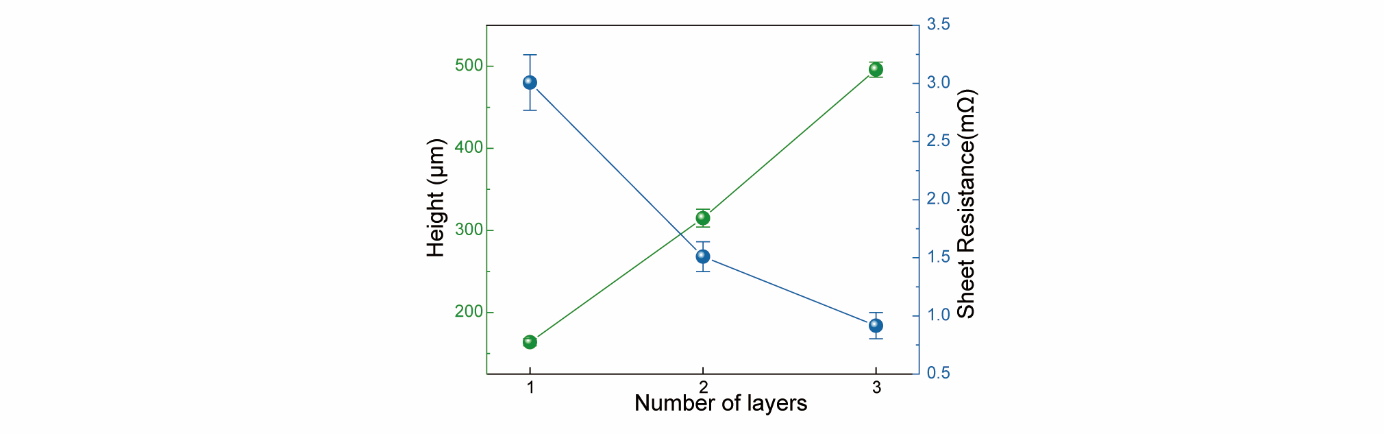


**Figure S32.** Layer-dependent variations in feature height and sheet resistance for printed tracks in NFC tags. Data represents mean ± s.d. (n = 3).


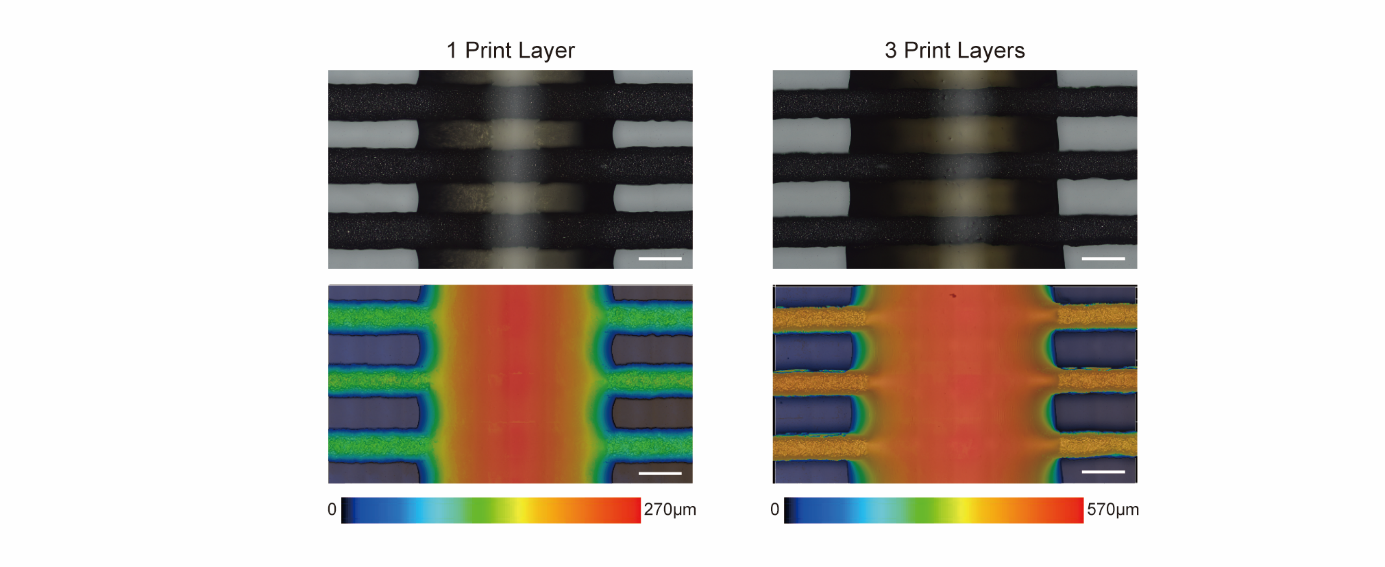


**Figure S33.** Optical and optical topographic images of loop antennas with different print layers that are covered by elastomer barriers for electrical insulation. Scale bars: 500 μm. The characterizations confirm that the elastomer barrier effectively encapsulates the underlying liquid metal composite tracks.


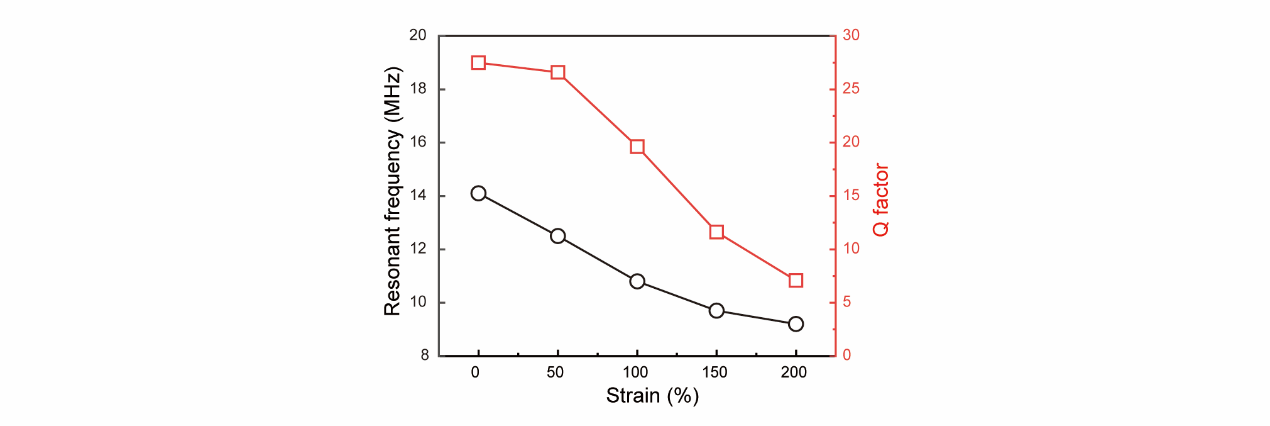


**Figure S34.** Resonant frequency and Q factor of the NFC tag at different tensile strains. Both the resonant frequency and Q factor decrease as the strain increases, due to the increased resistance and distorted shape of the loop antenna.


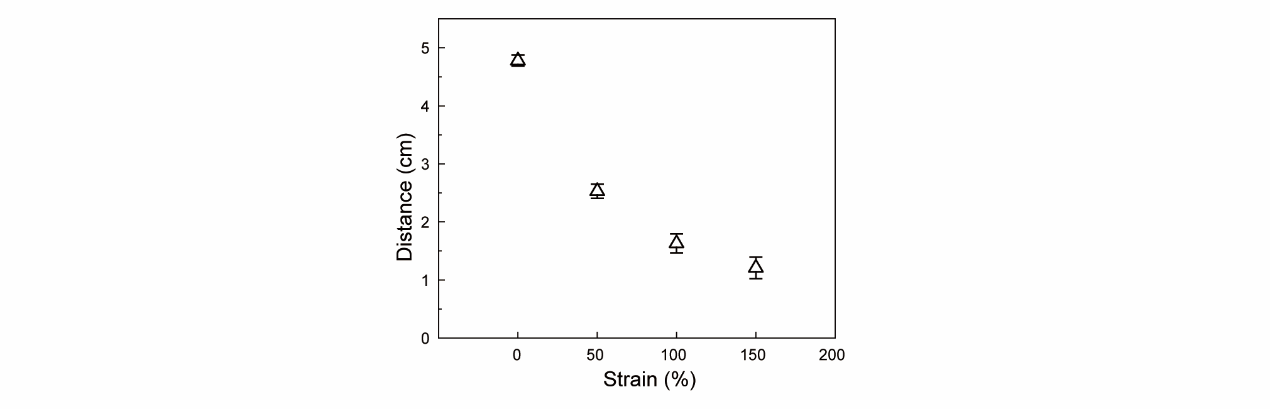


**Figure S35.** Maximum operation distance of stretched NFC tags used to unlock the door at various strain levels. The NFC tag loses its communication with the smart lock at 200% strain due to a significant shift in the resonant frequency. Data represents mean ± s.d. (n = 4).

**Supplementary Movies**

**Movie S1.** Generating a single-layer star pattern by 3D printing with liquid metal emulsion gels.

**Movie S2.** Generating a stacked star pattern by 3D printing with liquid metal emulsion gels.

**Movie S3.** Operation of an LED matrix display during biaxial stretching to 300% area strain.

**Movie S4.** Operation of an NFC tag affixed to the back of a hand in its relaxed state.

**Movie S5.** Operations of an NFC tag attached to the wrist under bending conditions.
